# Population genomics of Mediterranean oat (*A. sativa*) reveals high genetic diversity and three loci for heading date

**DOI:** 10.1007/s00122-021-03805-2

**Published:** 2021-03-26

**Authors:** F. J. Canales, G. Montilla-Bascón, W. A. Bekele, C. J. Howarth, T. Langdon, N. Rispail, N. A. Tinker, E. Prats

**Affiliations:** 1 Institute for Sustainable Agriculture, CSIC, Avenida Menéndez Pidal, 14004 Córdoba, Spain; 2 Ottawa Research and Development Centre, Agriculture and Agri‐Food Canada, Ottawa, ON Canada; 3 Institute of Biological, Environmental and Rural Sciences, Aberystwyth Univ, Aberystwyth, UK

## Abstract

**Key message:**

Genomic analysis of Mediterranean oats reveals high genetic diversity and three loci for adaptation to this environment. This information together with phenotyping and passport data, gathered in an interactive map, will be a vital resource for oat genetic improvement.

**Abstract:**

During the twentieth century, oat landraces have increasingly been replaced by modern cultivars, resulting in loss of genetic diversity. However, landraces have considerable potential to improve disease and abiotic stress tolerance and may outperform cultivars under low input systems. In this work, we assembled a panel of 669 oat landraces from Mediterranean rim and 40 cultivated oat varieties and performed the first large-scale population genetic analysis of both red and white oat types of Mediterranean origin. We created a public database associated with an interactive map to visualize information for each accession. The oat collection was genotyped with 17,288 single-nucleotide polymorphism (SNP) loci to evaluate population structure and linkage disequilibrium (LD); to perform a genome-wide association study (GWAs) for heading date, a key character closely correlated with performance in this drought-prone area. Population genetic analysis using both structure and PCA distinguished two main groups composed of the red and white oats, respectively. The white oat group was further divided into two subgroups. LD decay was slower within white lines in linkage groups Mrg01, 02, 04, 12, 13, 15, 23, 33, whereas it was slower within red lines in Mrg03, 05, 06, 11, 21, 24, and 28. Association analysis showed several significant markers associated with heading date on linkage group Mrg13 in white oats and on Mrg01 and Mrg08 in red oats.

**Supplementary Information:**

The online version contains supplementary material available at 10.1007/s00122-021-03805-2.

## Introduction

Oat is an important crop of Mediterranean origin that is used worldwide as food, feed grain, green fodder and as a winter cover crop in no-till rotations (Stevens et al. 2004). *Avena sativa* L. subsp. *sativa* and subsp. *byzantina* sometimes known as the white and red oat, respectively, are the main cultivated oats. They are grown most widely in temperate areas, with an increasing interest to expand the crop to subtropical areas and Mediterranean countries (Stevens et al. 2004). This is mainly due to its good adaptation to a wide range of soil types and because on marginal soils oats can perform better than other small-grain cereals (Stevens et al. 2004). Currently, the cultivated area of oats in the Mediterranean rim is equal to that of the northern European countries (Rispail et al. 2018). However, in Mediterranean areas, oat is sensitive to hot and dry weather due, in part, to a high transpiration rate (Ehlers 1989). Indeed, drought is the main limiting factor for oat yield, exceeding losses from all other causes under some conditions (Stevens et al. 2004). In addition, oat fungal diseases such as powdery mildew, caused by *Blumeria graminis* f.sp. *avenae,* and crown rust, caused by *Puccinia coronata,* are major constrains for this crop. The use of resistant varieties is one of the best control alternatives (Stevens et al. 2004). However, most of the current oat cultivars used for winter cropping in the Mediterranean rim are spring cultivars bred in northern countries that are not well-adapted to Mediterranean conditions (Prats et al. 2014). This might explain the poor oat adaptation observed in this area.

In contrast to the poor adaptation observed in many oat cultivars growing under Mediterranean conditions, landraces (for which this area is particularly rich) are regionally adapted to this environment. They provide a tangible gene pool for future genetic breeding programs and food security (Chalak 2015). Transfer of beneficial traits from landraces is relatively straightforward as there is no barrier for crossing. However, during the last century, the replacement of landraces by modern cultivars has led to a significant genetic erosion in many cereal crops (Newton et al. 2010). Studies on maize (Warburton et al. 2008) and wheat (Reif et al. 2005; Roussel et al. 2004) have identified a significant reduction in diversity. These studies also suggest that landraces may be a good source of new allelic diversity for breeding programs. Germplasm banks are facilities designed to provide long-term conservation of genetic resources including landraces. However, more exhaustive characterization of these collections, ranging from morphological or agronomical features to genome information, is necessary to assure maximum utilization of this germplasm. Moreover, association of genetic markers with regions of the genome controlling different traits would enable the development of marker-assisted selection for efficient and precise transfer of useful alleles from landraces to modern cultivars.

Genetic diversity, assessed by various tools including DNA markers, is important information both for genetic conservation and for the efficient breeding of new commercial varieties. DNA markers such as amplified fragment length polymorphisms (AFLPs), random amplified polymorphic DNA (RAPDs) or simple sequence repeats (SSRs or microsatellites) have been used to assess oat genetic diversity and to examine allelic diversity changes over 100 years of oat breeding in both Nordic countries and Canada (e.g., Fu et al. 2007). Recently however, genotyping-by-sequencing (GBS) has emerged as a more robust genomic approach to explore plant genetic diversity of species with complex genomes that lack extensive genomic resources such as oat (Elshire et al. 2011). GBS has been used to identify genomic regions associated with particular traits by bi-parental mapping (e.g., Kebede et al. 2020) or genome-wide association analysis (GWAS, e.g., Esvelt Klos et al. 2017).

In this work, we characterized 709 oat accessions, mainly consisting of Mediterranean red and white oat landraces, from 24 different countries. We created an interactive map to easily access their main morphological and passport information and we dissected their genetic relationships using a detailed genetic diversity study through GBS. Furthermore, we carried out a GWAS of heading date, a key character determining performance in this drought-prone area, that validates the strength of this collection and the genetic information developed.

## Materials and methods

### Plant material

A collection of 709 accessions of white and red *Avena sativa* L. was assembled, containing a majority of landraces from the Mediterranean area. Seeds were obtained from “Centro de Recursos Fitogenéticos” (INIA, Madrid, Spain) and United States Department of Agriculture (USDA, Washington, USA). Cultivars included within the collection were provided by various institutions as reported by Sánchez-Martín et al. (2014). They were included for comparison since they are widely cultivated in the Mediterranean area. All accessions were purified through single seed descent over three generations prior to genotypic and phenotypic evaluation.

### Phenotypic data

The Mediterranean oat collection was evaluated in three different environments in Spain for heading date. Trials were performed in two contrasting locations, Santaella (Spain), with 238 m altitude, and light clay eutric gleysol during growing season 2017–2018 and Córdoba (Spain), with 90 m altitude and light clay calcic cambisol during growing seasons 2016–2017 and 2017–2018. At each location-year, an alpha lattice square design with three replicates was used and the cultivar ‘Patones’ was used as check. The randomization of genotypes was done with SPSS v. 25 software (IBM Corp. Armonk, NY, USA). Each replicate consisted of 27 × 27 1-m-long rows containing the 709 genotypes plus additional checks included in each row and column until completing the lattice square. Rows were separated from each other by 30 cm, at a sowing density of 200 seeds m^−2^. Replications were bordered by cv ‘Flega’. Sowings took place in December, according to local practices. No irrigation was performed in the trials. Hand weeding was carried out when required, and no herbicides or fertilizers were applied. Phenotypic data are available in Online Resource 1.

### Interactive map

The interactive map with the 709 oat accessions was created with Google Maps (Google-LTD 2021). Each accession was located according to its site of origin taken from the passport data provided by the germplasm bank database. For each located accession, additional data were stored including (1) general information from its passport data, (2) agronomic information collected from field experiments joined by pictures of spikelets and seeds. In addition, different map layers were added where each variety can be visualized by spot color depending on subspecies (*byzantina*, *sativa* or admixture), heading date (early, mid or late heading), improvement status (cultivar, landrace or breeding material) and geographic elevation (low or high altitude).

### DNA extraction, library construction and sequencing

Leaf tissue was harvested from single plants growing in the field and lyophilized before DNA extraction. DNA was extracted according to the CTAB protocol (Murray and Thompson 1980) and the complexity reduced, multiplexed GBS libraries were constructed by Plateforme d’Analyses Génomiques of the Institut de Biologie Intégrative et des Systèmes (IBIS), Université Laval (Quebec City, Canada), based on the *Pst*I–*Msp*I method described by Huang et al. (2014). Complexity reductions were multiplexed using barcode adapters, with 96 samples per pooled library. Sequencing of each pooled library was performed on a single lane of a HiSeq 2500 platform (Illumina, San Diego, CA, USA) using standard Illumina protocols and kits, producing high-output paired-end 150 bp reads at the Genome Quebec sequencing center (Montreal, Canada).

### Tag‐level haplotype and SNP analysis

Raw sequence files in FASTQ format were processed using initial steps of the UNEAK-GBS pipeline within TASSEL 3.0 bioinformatics analysis package (Lu et al. 2013) to trim the reads, de-convolute the barcodes and produce a single tag count file for each sample. These files were then used by the Haplotag production pipeline (Tinker et al. 2016) to call marker genotypes (including both tag-level haplotypes and GBS SNPs) based on pre-determined genetic loci identified previously in a set of 4,657 *A. sativa* accessions reported by Bekele et al. (2018). The resulting data matrix was filtered using in-house software, to remove all markers with lower than 80% completeness, less than 5% minor allele frequency, or greater than 10% heterozygosity from the data matrix. Since the production mode was developed in a population that was dominated by North American germplasm, which might introduce ascertainment bias, a second dataset was created by running the Haplotag pipeline in discovery mode together with a representative set of North American landraces from the population reported by Esvelt Klos et al, (2016). These data were filtered using the same parameters as the genotype calls from the production mode. The genetic positions of the markers on the consensus map (Bekele et al. 2018) were identified. Structure and PCA analyses showed no differences in the population structure and clustering of the Mediterranean accessions between the production and discovery dataset. Therefore, all subsequent analyses were performed with the production dataset to take advantage of the marker map position except for the PCA analysis.

### Population structure and kinship

Population structure of the Mediterranean oat GBS dataset was inferred in the production mode matrix with 17,288 polymorphic GBS-SNP markers by the software STRUCTURE 2.3.4 (Pritchard et al. 2000) using the admixture model and the option of correlated allele frequencies between populations, as this configuration is considered best by Falush et al. (2003). Similarly, we let alpha (the degree of admixture) be inferred from the data. Each simulation included 10,000 burn-in iterations, 20,000 iterations and ten independent simulations per *k* value. The number of subpopulations and the percentages of admixture of each accession (*Q* matrix) were given by STRUCTURE HARVESTER (Earl and vonHoldt 2012), an online software to visualize STRUCTURE output implementing the Δ*k* method (Evanno et al. 2005). For subsequent analyses, accessions were assigned to a subpopulation when they showed more than 60% membership in a subpopulation. Visualization of STUCTURE data was done with the online software STUCTUREPLOT (Ramasamy et al. 2014).

Principal component analysis (PCA) was also performed as an alternative method to infer the structure of the collection with the software package TASSEL 5 (Bradbury et al. 2007). In addition, two UPGMA dendrograms of white and red oat accessions were computed using TASSEL 5 with default settings (Bradbury et al. 2007). The estimated trees were then drawn with the ggplot2 package in R (R-Team, 2017). Accessions were labeled with different colors according to their geographic origin (north Mediterranean, west Mediterranean, south Mediterranean and east Mediterranean).

### Linkage disequilibrium

Pairwise measures of LD based on *r*^2^ were computed using TASSEL 5 for the whole collection of Mediterranean oat as well as for each oat subspecies (*sativa* and *byzantina*). LD test was calculated for each marker pair (full matrix) and per chromosome to assess the overall linkage disequilibrium of the collection and the distance of linkage decay per linkage group. The LD decay plots were generated by using locally estimated smoothed lines (LOESS) (Jacoby 2000) in R (R-Team, 2017).

### Genome‐wide association mapping for heading date

Marker-phenotype associations were estimated with the software package TASSEL 5, according to Rispail et al. (2018). Associations were tested using a general linear model with PCA covariates (GLM-PCA) and with a mixed linear model (MLM) using the PCA coordinates for *Q* and the kinship distances for *K*, as proposed by Yu et al. (2006). Models were run using mean heading dates across the three environments as phenotypes. Mean heading date values for each genotype within an environment were normalized according to their respective checks. Combined quantile–quantile plots drawn to compare the observed distribution of – log_10_(*P*) values for each marker-trait association using the cumulative distribution to assess the correct fitting of the data and the control for type I error. Selection of the optimal model was performed by estimating the genetic inflation factor *λ*median, as follows: *λ*median = median (Sc + 1, Sc + 2, …, Sn)/0.456. A genetic inflation factor higher than 1.25 (*λ* > 1.25) indicates data stratification even after structure corrections. Therefore, *p* values for all models with *λ* > 1.25 were adjusted with their respective λ to avoid false positives (Reed et al. 2015). The critical adjusted *p* values for assessing the significance of associations were corrected for multiple testing based on the false discovery rate (FDR) criteria (Benjamini and Hochberg 2000). The matrix of *p* values was used to estimate the corresponding *q* values with the *Q*value package (Storey 2002) in R (R-Core-Team, 2017. Manhattan plots of GWAS results were drawn with the qqman package in R (R-Team, 2017).

### Sequence homology

We used GBS markers with known genetic position on the oat consensus map (Bekele et al. 2018) to find nearby annotated genes in two sequenced diploid oat genomes (*Avena atlantica* (AA) and *Avena eriantha* (CC) described by Maughan et al. (2019) and in a recently released pseudomolecule assembly of hexaploid oat from the Canadian breeding line ‘OT3098’ (available July 2020 at https://wheat.pw.usda.gov/GG3/node/922). GBS marker positions in the diploid genomes were declared based on the best BLAST match of the reference sequences of the GBS markers. In the hexaploid genome, GBS positions were declared when an exact match of a 64-base tag-level haplotype belonging to a given locus was found at one and only one genome position. At the time of submission, the finalization of hexaploid chromosome names and orientations was still before the Nomenclature Committee of the International Oat Conference, thus we refer to chromosome identities and genome positions as “Hexaploid Oat Reference OT3098 V1”. Online Resource 2 shows a working correspondence between Mrg consensus groups and chromosome identities in the above diploid and hexaploid pseudomolecules based on the best match of ten random markers for each linkage group. The function BlastX of the BLAST algorithm (Altschul et al. 1990) was used to validate protein identities in the NCBI non-redundant protein database (database released on 27 Julio 2020), as implemented in the NCBI webserver (http://blast.ncbi.nlm.nih.gov/Blast.cgi).

## Results

### Characterization of the oat collection and localization in an interactive map

**Fig. 1:**
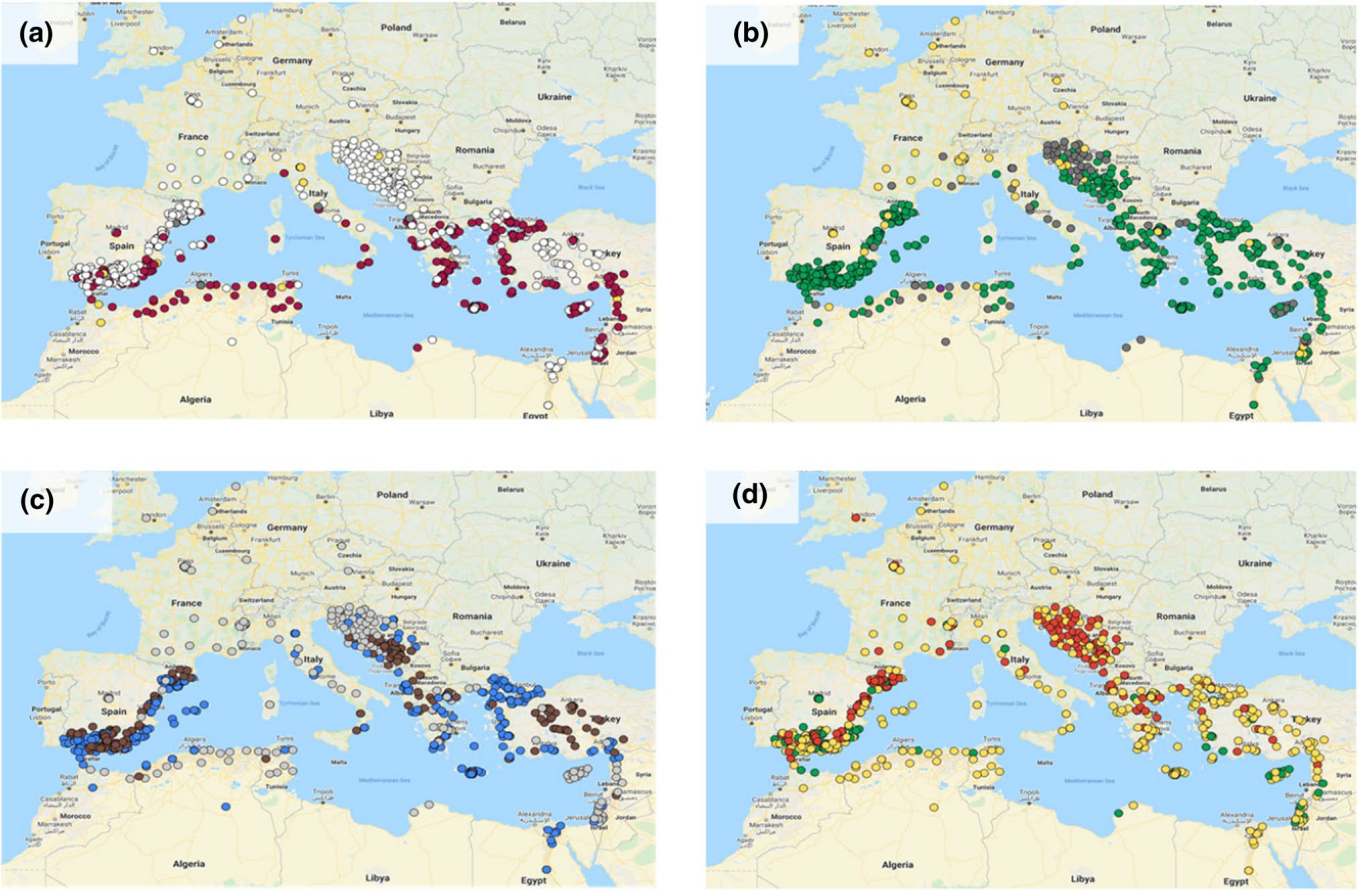
Example of the interactive oat map as can be seen in google maps. In this example, the accessions were filtered according to **a** subspecies where white, red, yellow and gray dots indicated *sativa*, and *byzantina* subsp, admixture and unknown, respectively, **b** improvement status, where green, yellow, purple and gray dots indicated landraces, cultivars, breeding materials and uncertain materials, respectively, gray dot indicated that information is not available, **c** altitude where brown, blue and gray dots indicated landraces collected in high (> 400 m) or low (< 400 m) altitude regions, cultivars and other accessions for which altitude was not available were represented by gray dots, **d** heading date, where green, yellow and red dots indicated early, mid or late flowering accessions

Data from the accessions of the oat Mediterranean collection including the north-European cultivars are shown on the interactive map (https://www.google.com/maps/d/viewer?mid=1t-O7OUoUPJ_qY5qGQsq66O5zg7k) where the different classification criteria may be used as filters yielding several layers that show a different color code according to the selected criteria. The data underlying the map are also available at Dryad Data (Canales et al. 2021; https://doi.org/10.5061/dryad.0gb5mkm0g). Different layers of the map (e.g., Figure 1), showed approximately even representation across most classifications other than improvement status, where the majority of accessions (546 of 709) were landraces (Fig. 1b). The oldest landrace was accession 370, which is a landrace from Evros in Greece collected in 1904. Most landraces were collected between 1940 and 1970, albeit some recently collected landraces were also included, such as accessions 245 and 246, collected in Balearic island, Spain, in 2009. Interestingly, some of the cultivars are even older than the landraces, including cv ‘Selma’ from Germany and ‘Joanette’ from France, both white oats from 1889. A total of 251 red and 435 white oats were included in the collection. As observed in Fig. 1a, both subspecies were collected from all around Mediterranean rim. There were accessions representing low- or high-altitude sites (Fig. 1c) and covering a wide range of heading dates (Fig. 1d). In addition to the main classification criteria, other passport data as well as detailed pictures of the spike morphology and seed morphology, color and size are available by clicking on the corresponding accession (e.g., Online Resource 3, 4).

### Genetic relationships among Mediterranean oats

The Haplotag production pipeline provided data for 164,652 tag-level haplotypes and 240,958 SNPs. After the filtering based on MAF, heterozygosity and missing values, a total of 17,288 polymorphic SNP markers corresponding to 12,418 tags were obtained. Of these tags, 4,829 were located on the hexaploid consensus map. Sequence read data from these accessions are available from NCBI SRA archive as BioProject PRJNA693576. The GBS-SNP marker datasets were deposited in the public T3/oat database (https://oat.triticeaetoolbox.org/breeders/trial/4667) and on the Dryad Data site (Canales et al. 2021, https://doi.org/10.5061/dryad.0gb5mkm0g).

**Fig. 2:**
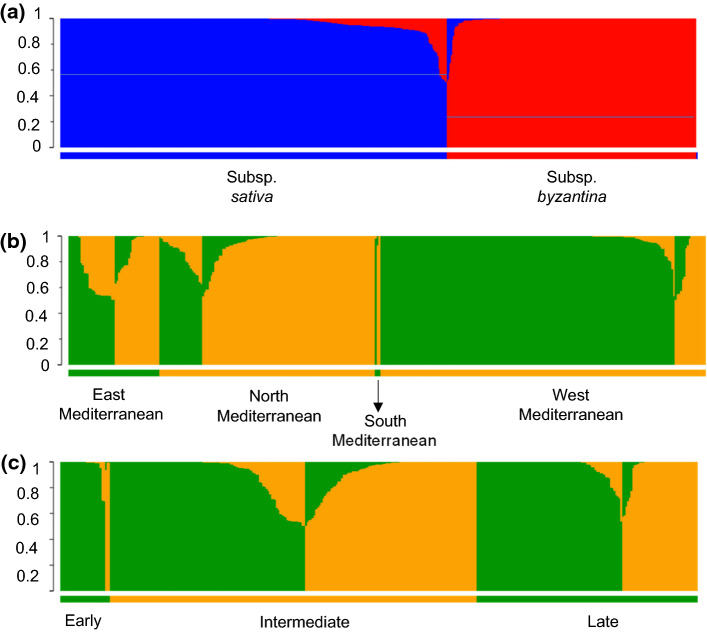
Estimated population structure of oat accessions according to STRUCTURE software. **a** Estimated population structure of the whole Mediterranean oat collection. The two colors indicate the two subpopulations in which STRUCTURE software classified the oat accessions, with the admixture barplot showing the admixture proportions of each individual. **b**, **c** Estimated structure of the white oat subpopulation, according to origin and flowering time

According to the position of the breaking point in the *L*(*k*) curve and the peak in the Δ*k* distribution, accessions were assigned by STRUCTURE to two groups (*K* = 2). This binary classification distinguished between the white (subsp. *sativa*) and the red (subsp. *byzantina*) oats (Fig. 2a). Out of the 709 accessions, 435 accessions were clustered in the white group, 251 accessions in the red group, 13 were classified as admixtures, and 10 showed a high level of missing data or heterozygocity and were not classified. Of the 699 classified accessions, only 28 assignments disagreed with prior passport data, and these are most likely due to previous classification errors. Based on passport data, it appears that admixtures are more common among red oat accessions than among white oats (Fig. 2a

The high genetic divergence between white and red oats may mask further population structure within groups. Therefore, separate analyses were performed on each group. Both the white and red oat UPGMA tree showed additional clustering when performed separately (Online Resource 6, 7). STRUCTURE analysis within the white group revealed two subgroups (Fig. 2b and c). Interestingly, most of the west-Mediterranean accessions, the majority of which were Spanish, were in subgroup 1, whereas most of the north-Mediterranean accessions were in subgroup 2. However, half of the east-Mediterranean accessions and a small part of north-Mediterranean accessions were in subgroup 1. Both subgroups contained accessions with a similar range of heading dates. However, further inspection of the UPGMA dendrogram showed that many of the west- and north-Mediterranean accessions tended to cluster based on heading date while those from the east- and south-Mediterranean accessions did not.

**Fig. 3:**
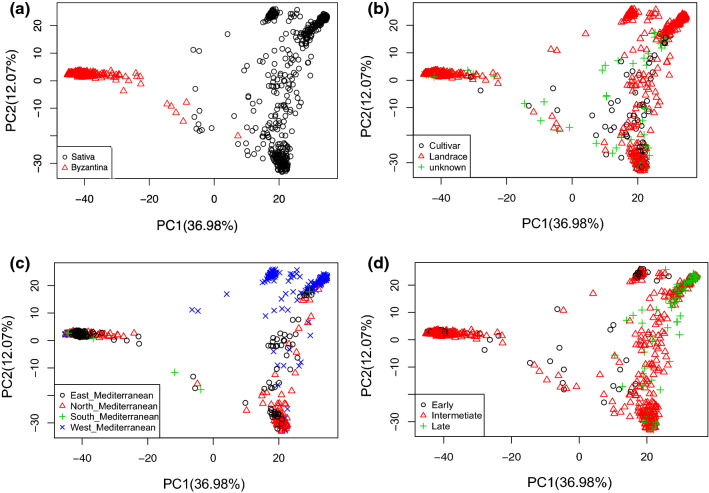
Scatterplot of principal component analysis scores of components 1 and 2 based on the 17,288 polymorphic SNP markers used in this study. The different PCA graphs depict **a** subspecies, **b** type of breeding material, **c** regions of origin and **d** flowering time

To explore the potential biological drivers of subpopulations, we performed PCA analysis (Fig. 3), highlighting different variables that could explain the splitting of these populations. In agreement with STRUCTURE analysis, PCAs separated the white and red oat groups (Fig. 3a). The first two PCs explained approximately 50% of the observed genetic variance. Interestingly, cultivars were distributed among both red and white groups and also within both subpopulations of the white group (Fig. 3b). Most of the west-Mediterranean accessions clustered in the upper part of the PCA plot in three smaller subgroups, whereas most of the north-Mediterranean accessions clustered in the lower part of the figure with a small group of east-Mediterranean accessions (Fig. 3c). Figure 3d shows that the clustering of the three smaller groups of west-Mediterranean accessions could be explained based on heading date, with one group showing most of the early accessions, another with most of the late accessions and the middle group with mixed or intermediate heading dates. In addition, most of the accessions that clustered in the lower part of the plot had late or intermediate heading dates. Interestingly, overall no red accessions were late, confirming the earliness of this subpopulation.

**Fig. 4:**
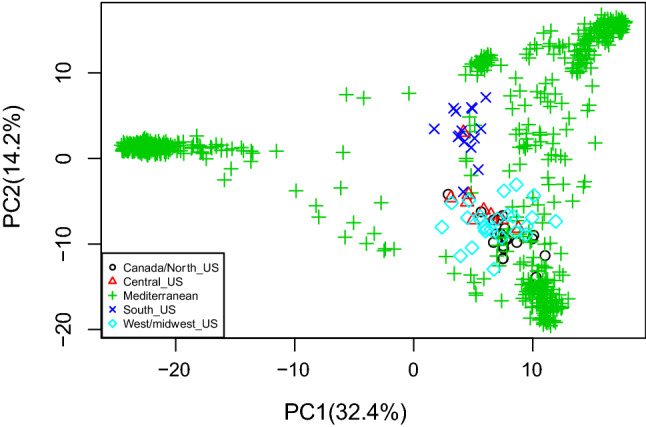
Scatterplot of principal component analysis scores of components 1 and 2 of Mediterranean and 69 North American oat accessions based on 20,493 polymorphic SNP markers

We further compared the genetic relationships of the Mediterranean accessions with a stratified diverse subset of 69 North American landraces previously characterized by Bekele et al. (2018). For this, we used *de-novo* SNP discovery to avoid potential ascertainment bias. After filtering, 20,493 polymorphic SNP markers were available for this analysis. Clustering of Mediterranean accessions obtained with this dataset was identical to the clustering obtained with the dataset from the production pipeline. Based on PCA analysis of these data (Fig. 4), we observed that North American landraces clustered in two groups corresponding approximately with the two subgroups of Mediterranean white oat. The southern USA landraces clustered nearest to the west-Mediterranean accessions, in particular the early heading Spanish group, whereas the remaining North American accessions clustered with the north-Mediterranean accessions (Fig. 4). In an additional analysis, we performed the PCA after downsampling Mediterranean oats to equal the number of US oats, to rule out possible artifacts due to group size and obtained similar results (Online Resource 8). Overall, this analysis revealed a high level of diversity in the Mediterranean oat collection when compared to the North American accessions.

### Genome-wide association analysis of heading date

**Fig. 5:**
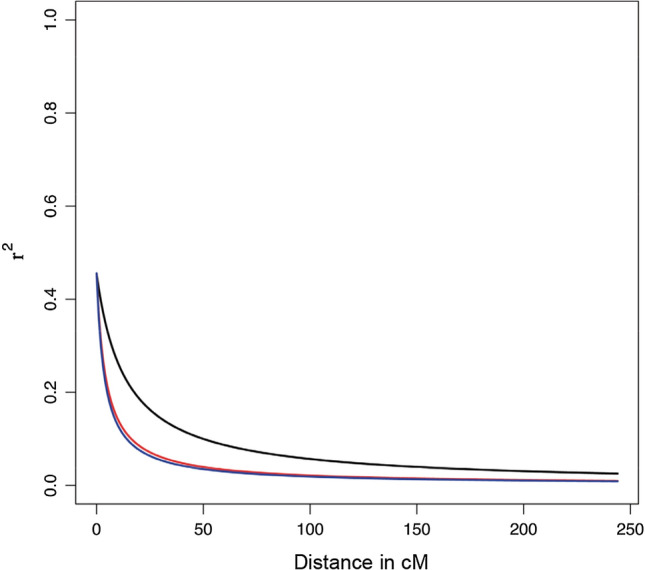
Linkage disequilibrium (LD) decay by distance across the subspecies *sativa* (blue line), *byzantina* (red line) and both subspecies (black line) (color figure online)

Assessment of LD is critical for association analysis since it may determine the power and resolution of associations. The patterns of LD were assessed by pairwise comparison of markers presented as the relationship between *r*^2^ *vs.* *P* (Online Resource 9). The rate of LD decay against genetic distance was calculated for total genome data (Fig. 5) as well as for each linkage group (Online Resource 10).

According to the full LD matrix, a total of 9,298,006 marker pairs showed a significant LD, with an average probability of *p* = *0.006.* Of these, 5,773,494 marker pairs showed *r*^2^ < 0.1, which is considered a nominal value for oats (Montilla-Bascón et al. 2015; Newell et al. 2011). When the two subspecies were assessed separately, 2,869,144 markers pairs showed a significant *r*^2^ < 0.1 within white oats, whereas a much lower number of marker pairs (99,327) showed a significant *r*^2^ < 0.1 within red oats (Online Resource 9).

Both the red and the white groups showed a similar LD decay, but the decays were faster than that across the full population (Fig. 5). Inspection of the LD by linkage groups showed differential trends. In white oat, several linkage groups showed higher LD decay including Mrg03, 05, 06, 11, 21, 24 and 28, whereas in red oat LD decayed faster on Mrg01, 02, 04,12, 23 and 33. Strikingly, linkage group Mrg04 showed a much slower LD decay in white oat than in red oat or in the combined population. The remaining linkage groups showed similar LD decay for white and red oats (Online Resource 10).

**Fig. 6:**
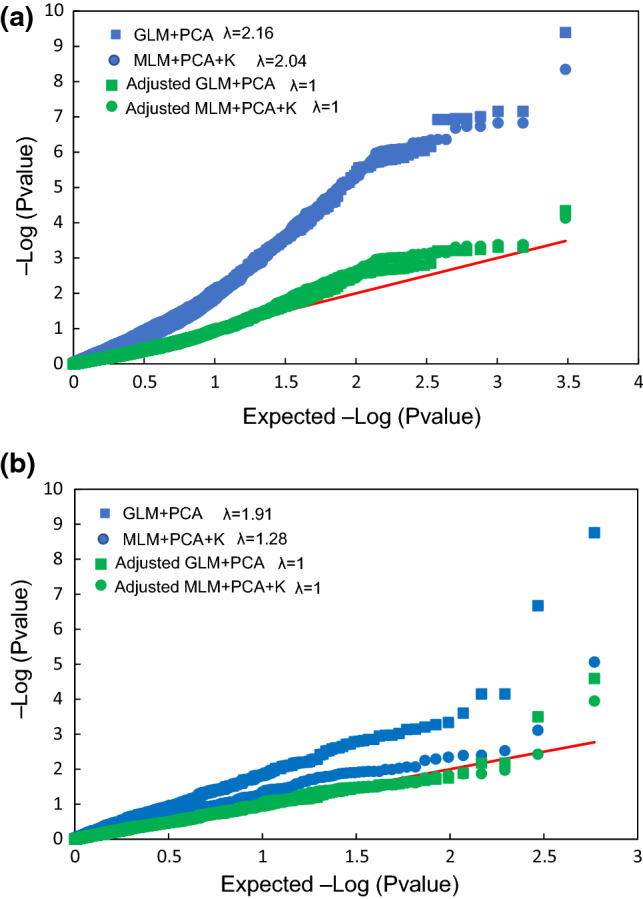
Distribution of *p* values for the different models used in this study for the association between markers and heading date in the **a** white oat accessions and **b** red oat accessions. Axes represent the expected *p* values versus the observed *p* values in the negative log_10_ scale, where the solid red line represents the null expectation (absence of type I error). The genetic inflation factor (*λ*) was used for correction of the models (color figure online)

**Fig. 7:**
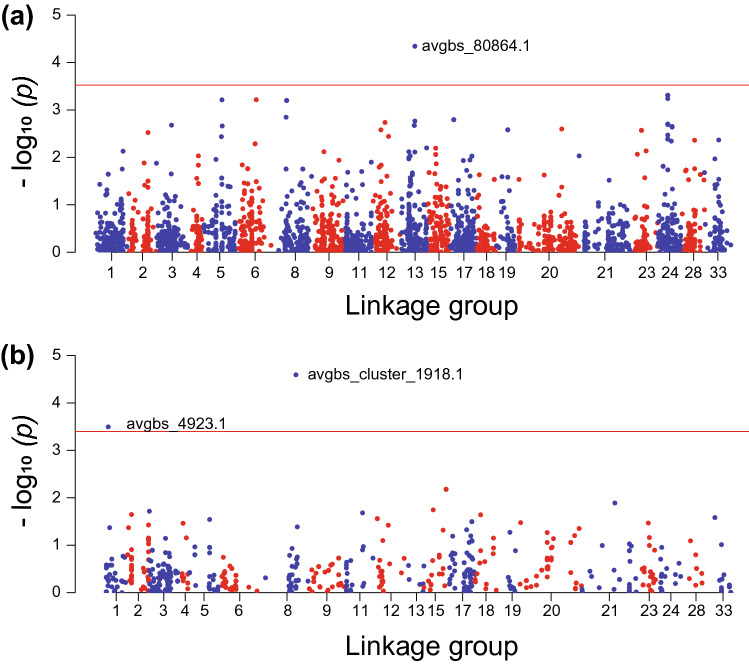
Distribution of *p* values by marker position on chromosomes for tests of association between markers and heading date using the adjusted GLM model in the **a** white oat accessions and **b** red oat accessions. Red and blue colors show divisions between linkage groups. The solid red line represents the genome-wide significance threshold calculated according FDR (color figure online)

Due to the strong population structure, association studies were performed separately for white and red oats. Despite the fact that both models were corrected for population structure, data for white oat did not fit either the GLM-PCA or the MLM models. Increasing the numbers of PCs up to 20 in the GLM-PCA and 10 for MLM data improved the fit of these models, but the genetic inflation factor was still far from the proposed *λ* = 1.2 threshold (Fig. 6a, Rispail et al. 2018). To avoid false positives, we adjusted the models based on the genetic inflation factor, which resulted in a much better fit (Fig. 6b). Following this, one significant marker associated with heading date was found within white oat. This marker, avgbs_80864.1, was located in Mrg13 at cM position 60. It was significant in both the GLM-PCA and MLM models with an adjusted of *P* = 7.3e^−5^ and an *r*^2^ = 0.103. Both GLM-PCA and MLM models revealed two significant associations with marker avgbs_cluster4923.1 located on Mrg01 at cM position 52 (Fig. 7) with an adjusted of *P* = 3.18e^−4^ and *r*^2^ = 0.044 and marker avgbs_cluster_1918.1 located on Mrg08 at cM position 147 (Fig. 7) with an adjusted of *P* = 2.56e^−5^ and *r*^2^ = 0.069.

### Identification of candidate genes

To identify potential candidate genes involved in heading date, we located the markers onto the diploid (Maughan et al. 2019) and hexaploid (https://wheat.pw.usda.gov/GG3/node/922) oat reference genomes and examined the genomic space surrounding each associated marker using the Gbrowse tool implemented in the EPIC-CoGe Avena genome repository (Table 1, Online Resource 11). The marker avgbs_80864.1 (on Mrg13) was localized to the C genome chromosome AE05 in *A. eriantha* at position 463,706,207 bp and to position 472,093,139 bp on chromosome 2C in the hexaploid. The closest gene in the diploid, named AE025378-RA, was detected at 10 kb downstream of the associated marker. This gene showed similarity with the NAC domain-containing protein 100 (NAC100) of *Arabidopsis thaliana* which was validated by BLASTX (Table 1, Online Resource 11). Additional genes in the vicinity of avgbs_80864.1 marker were located more than 100 kb away. Annotated genes in the hexaploid genome were not located in this region, and although BLAST matches can be located, many of these appear to be repetitive domains, and further gene discovery in the hexaploid will require further annotation.

**Table 1:** Potential candidate genes of significant markers in two sequenced diploid oat genomes, *Avena atlantica* (AA) and *Avena eriantha* (CC) described by Maughan et al. (2019) using the function BlastX of the BLAST algorithm (Altschul et al. 1990). Blastx 2.2.30

Marker	Linckage group	Chromosome	Nearby gene	Gene/marker distance (kbp)	Position relative to marker	Results BLASTn NCBI					
						Description	Species	*E*-value	Cov (%)	Ident (%)	Accession number
avgbs_80864.1	Mrg13	A. eriantha AE05	AE025378-RA	10	Downstream	NAC domain-containing protein 92-like	*A. tauschii*	0.0	99	83.38	XM_020312824.1
			AE025381-RA	319.84	Downstream	30S ribosomal protein 3, chloroplastic	*B. distachyon*	8E-152	17	87.42	XM_003579859.4
			AE025377-RA	24.98	Upstream	Cyclic pyranopterin monophosphate synthase, mitochondrial	*B. distachyon*	0.0	21	90.88	XM_024456111.1
			AE025376-RA	45.6	Upstream	Gamma gliadin, omega gliadin-B6, LMW-B2, and LMW-B3 genes	*T. aestivum*	0.0	68	78.77	MG560141.1
			AE025375-RA	77.13	Upstream	Vacuolar amino acid transporter 1-like	*A. tauschii*	0.0	72	88.28	XM_020312820.1
avgbs_cluster_4923.1	Mrg01	A. atlantica AA02	AA010050-RA	27	Downstream	Phosphatidylinositol N-acetylglucosaminyltransferase subunit P-related	*Z. mays*	0.0	85	74.18	XM_008659956.2
			AA010051-RA	33	Downstream	Rop guanine nucleotide exchange factor 3-like	*H. vulgare*	0.0	97	90.13	KAE8786664.1
			AA010053-RA	46.344	Downstream	Pectin acetylesterase 9	*B. distachyon*	6E-65	21	81.85	XM_024460397.1
			AA010057-RA	147.586	Downstream	Proline dehydrogenase 2, mitochondrial-like	*A. tauschii*	0.0	60	93.93	XM_020328890.1
			AA010060-RA	170.425	Downstream	GDSL esterase/lipase At5g55050-like	*A. tauschii*	2E-29	1	83.12	XM_020305331.1
			AA010048-RA	193.935	Upstream	Branched-chain amino-acid aminotransferase 5,	*A. tauschii*	0.0	100	91.06	XM_020325288.1
avgbs_cluster_1918.1	Mrg08	A. eriantha AE06	AE037406-RA	0.75	Downstream	NAC domain-containing protein 8	*H. vulgare*	0.0	97	84.32	KAE8770891.1
			AE037407-RA	133.402	Downstream	Histone H2B.1-like	*A. tauschii*	0.0	72	95.5	XM_020293063.1
			AE037408-RA	144.59	Downstream	Clone B1 GASR7 gene	*T. aestivum*	6E-75	17	94.74	KJ000053.1
			AE037414-RA	251.266	Downstream	Transcription factor TGAL3-like	*A. tauschii*	1E-96	29	90.61	XM_020299922.1
			AE037405-RA	15.178	Upstream	Autophagy-related 2	*A. tauschii*	0.0	50	88.36	XM_020305375.1

GBS marker positions in the diploid genomes were declared based on the best BLAST match of the reference sequences of the GBS markers

The avgbs_cluster_4923.1 marker (Mrg01) was localized to chromosome AA2 of *A. atlantica* at position 364,214,519 bp and to chromosome 1D at 249,647,335 bp in the hexaploid. Two potential candidate genes were detected downstream from this marker in the diploid. One, AA010050-RA, located 27 kb upstream and the other, AA010051-RA, at 33 kb downstream. According to BLASTX comparison, AA010050-RA shared more than 70% similarity with a Phosphatidylinositol N-acetylglucosaminyltransferase subunit P-like protein of *Zea mays*. In turn, AA010051-RA encodes a Rop guanine nucleotide exchange factor 3 (ROPGEF3), as confirmed by the BLASTX showing a similarity of 90.13% and 55.41%, respectively, with ROPGEF3 homologues in *Hordeum vulgare* and *Arabidopsis thaliana* (Table 1, Online Resource 11). Three additional genes were also identified between 40 and 50 Kb downstream of the associated marker, one with homology to a pectin acetylesterase and two unknown proteins. The closest upstream gene, had unknown function and was located more than 140 kb away.

The associated marker avgbs_cluster_1918.1 on Mrg08 was localized on *A. eriantha* genome to chromosome AE06 at position 326,942,176 bp and to chromosome 1D at 249,647,335 bp in the hexaploid. One potential candidate gene was identified only 0.75 kb downstream of this marker in the diploid. This gene, AE037406-RA, is likely to encode another NAC-related gene since it shares 84.32 and 50.22% similarity with *Hordeum vulgare* and *Arabidopsis thaliana* NAC8, respectively (Table 1).

## Discussion

To our knowledge, this is the first large-scale genomic analysis of red and white oat landraces originating from the Mediterranean region. This region is the center of origin for many crop species and their wild relatives, including cultivated oat (Loskutov 2008), and oat has been cultivated in this region for centuries. Hence, this work will provide a valuable resource for future research and crop improvement worldwide.

Over the last two decades, the increasing worldwide cultivation of elite cultivars has led to a significant reduction in genetic diversity of crop species (Warburton et al. 2008; Reif et al. 2005; Roussel et al. 2004) particularly in cereals (Christiansen et al. 2002; Donini et al. 2000; Koebner et al. 2003). This highlights the role of the genetic conservation of crop germplasm (Frankel and Bennett 1970). Fortunately, genebanks are available as reservoirs of crop genetic diversity. However, genebanks contain duplicated accessions, errors in classification and omissions of key data that would guide plant breeders in the utilization of this germplasm. In particular, many genebank accessions lack data related to phenotype or imagery that could allow correction of classification errors. Thus, this work gathered a wealth of information about the oat genetic and phenotyping diversity of oat along the Mediterranean rim, which could be used to correct the genebanks database errors. The interactive map presented in this work provides an essential resource for future oat breeding for Mediterranean areas, by which oat breeders may select accessions taking into account additional agronomic and geospatial data. The map has been conceived as a living tool in which further information developed on these accessions will be added, including disease and abiotic stress resistance, to facilitate oat breeding under a scenario of climate change. Where possible, we have updated or corrected classifications made by genebanks such that the interactive map includes new information consistent with the genetic analyses presented here. When subspecies classification differed from that of the genebank this was highlighted in the map information, so genebanks can check it if desired.

This work has revealed high genetic and phenotypic variability and a complex population structure in a Mediterranean oat collection. The GBS data provided high-density, genome-wide and genetically mapped marker set. Based on the Bayesian STRUCTURE analysis, the Mediterranean oat landraces clustered with high confidence into two subpopulations, corresponding to the *sativa* and *byzantina* subspecies, with higher genetic variability and more subpopulation structure in the *sativa* group. This was also observed in a previous genetic diversity study on Spanish accessions (Montilla-Bascón et al. 2013) suggesting that red oats have been less subjected to population divergence than white oats. As previously reported, we found a significant genetic divergence between white and red oats (Fu et al. 2005; Newell et al. 2012), supporting the independent domestication hypothesis of these two subspecies (Zohary and Hopf 2000). While the Fertile Crescent is considered the center of origin of *A. sativa,* it is argued that white oat evolved during the migration of its ancestors northward, where it was eventually established as a primary crop (Thomas 1995). In contrast, red oats entered cultivation from the western part of the Mediterranean (Loskutov 2008).

Interestingly, the oat cultivars included in the collection for comparison purposes clustered nearer to the north- and east-Mediterranean landraces than to the west-Mediterranean landraces. This could explain the low adaptation of most of the cultivars currently grown in Spain, the majority of which are spring cultivars bred in northern areas (Prats et al. 2014; Rispail et al. 2018; Sánchez-Martín et al. 2017). Our previous study based on 36 north-European cultivars indicated a narrow genetic basis among the tested varieties. Similarly, a lack of genetic diversity has been reported in a collection of Canadian and Chinese oat cultivars, leading to a call for broadening the genetic variation for a sustainable oat improvement (Baohong et al. 2003; Fu et al. 2004). In contrast, the current Mediterranean oat collection showed no tight clustering of cultivars. Although cultivars did not cluster near to Spanish accessions as stated above, they were distributed over the PCA plot near landraces from various regions and distributed between the white and red oat clusters. This pattern suggests that different landraces may have contributed to the improvement of these cultivars and broadened their genetic base.

Adding the data gathered previously on a set of North American oat accessions to our data confirmed the high level of diversity in the Mediterranean collection. It also provided interesting information on the potential breeding history of oat accessions. North American oat landraces clustered primarily with the white oat landraces, suggesting that they had not significantly differentiated after migration to the new world, and that they had integrated little or no red oat germplasm. The fact that southern USA landraces grouped with the western Mediterranean landraces, most of which coming from Spain, may reflect both human migration patterns, as well as an adaptation to a Mediterranean-type production regime.

Population structure and LD are primary obstacles to the successful identification of linkage-based associations between markers and phenotypic traits (Buckler and Thornsberry 2002). Indeed, the power of association studies depends on the LD between the functional allele responsible for the observed phenotype and the marker. Our data showed extensive LD among markers, in agreement with previous studies (Montilla-Bascón et al. 2015; Rispail et al. 2018) and a sufficient marker coverage for a GWAS approach (Newell et al. 2011). Furthermore, our results revealed a variable LD pattern in the different linkage groups that was unique to each of the *sativa* and *byzantina* groups. Such patterns have also been found for other crop species such as maize, wheat and sugar beet (Li et al. 2011; Van Inghelandt et al. 2011). Interestingly, Mrg04 showed a much slower LD decay in subs. *sativa* than in *byzantina*. According to Alheit et al. (2012), regions containing strong LD blocks probably harbor QTL responsible for agronomically important traits that reduce LD decay. Thus, our data suggests the presence of QTLs responsible for agronomically important traits in this region, but only in subsp. *sativa*. In addition, according to Chao et al. (2010), this divergence in the extent of LD might be attributed to unique breeding histories and selection pressures applied to genes located in different genomes/chromosomes during the process of cultivar development.

Based on LD and structure results, association studies were performed separately for the two subspecies. The GWAS analysis was performed to test the suitability of the GBS approach and the Mediterranean oat collection for association studies by identifying significant associations with heading date. This trait is of fundamental importance for the local adaptation of oats in Mediterranean environments, since oats are particularly susceptible to drought during flowering time and heading date plays an important role for drought avoidance responses (Mahadevan et al. 2016). Our data highlighted markers associated with heading date on three chromosomes. Associations on these chromosomes have been previously reported on Mrg1 at 117.9 cM (Tumino et al. 2016) and at 39.3 cM (Esvelt Klos et al. 2016), on Mrg8 at 71 cM (Bekele et al 2018) and on Mrg13 at 58.6 cM (Tumino et al. 2016) and at 30.3, 33 and 35.9 cM (Esvelt Klos et al. 2016).Specifically, the associated marker that we identified on Mrg13 was only 1.4 cM apart from the QTL highlighted by Tumino et al. (2016), further validating the presence of a QTL for heading date in this region. By contrast, the markers identified on Mrg1 and Mrg08 were located in different regions than those reported previously suggesting the presence of additional QTLs. Interestingly, (Bekele et al. 2018; Esvelt Klos et al. 2016; Tumino et al. 2016) highlighted markers associated with heading date in Mrg02, which were not found in our study. This differences might be due to the different photoperiod during flowering for autumn and spring sowed oats.

Interestingly, two of the associated markers were located near genes with high similarity to NAC-domain transcription factors that have been involved in many developmental processes, including flowering. In particular, the closest gene on Mrg13 showed similarity to a NAC domain-containing protein 100 (NAC100), a transcription factor than controls ethylene-regulated cell expansion in flower petals (Pei et al. 2013). The closest gene to Mrg08 marker showed high homology with a suppressor of gamma response 1 (SOG1 / AtNAC8), which is a transcription factor governing multiple responses to DNA damage (Yoshiyama et al. 2009) and showing high expression in Arabidopsis thaliana during flowering (Klepikova et al. 2016). Comparison of the Mrg08 region between the *A. byzantina* and *A. sativa* preliminary genome assemblies also identified as potential candidate genes a GASA4-like gibberellin responsive gene and a gene with homology to miR172. Among the genes near the Mrg01 associated marker, one showed similarity with a phosphatidylinositol N-acetylglucosaminyltransferase subunit *P*, a protein in biosynthesis that is required for pollen germination and pollen tube growth (Kouidri et al. 2018; Lalanne et al. 2004). At this point, the influence of these genes on the time of heading is speculative, requiring further validation. Perfect GBS matches for all three of the high-confidence associations were also located in the recently released hexaploid oat genome. Unfortunately, a fully annotated version of this genome is not yet available so gene matches are tentative, and the reported genome positions may be interpreted at a later date. It is interesting to note, however, that the matches in the diploid genomes may not be located on the corresponding hexaploid chromosomes. For example, avgbs_cluster_1918.1 is located on a C genome chromosome in *A. eriantha*, while it is on a D genome chromosome in the hexaploid. Oat is known to have undergone extensive genome rearrangement, and forthcoming studies will elucidate the locations of some of these rearrangements.

In conclusion, this work has provided a wealth of genetic and agro-climatic information in Mediterranean landraces and cultivars of oat. These accessions, genetic data and phenotypes will be a vital resource for further discovery-based research as well as for global oat genetic improvement.

## Supplementary Information

Below is the link to the electronic supplementary material.

Supplementary file1 (MP4 10926 kb)

Supplementary file2 (PDF 2582 kb)

## Declarations

### Availability of data and material

Genotype data were deposited in the public T3/oat database (https://oat.triticeaetoolbox.org/breeders/trial/4667). GBS FASTQ raw data have been submitted to the NCBI SRA (Bioproject PRJNA693576). Interactive map with passport and phenotyping data of the accessions is available at (https://www.google.com/maps/d/viewer?mid=1t-O7OUoUPJ_qY5qGQsq66O5zg7k. The data underlying the map and also marker data have been submitted to the DryadData and are accessible as Canales et al. 2021, https://doi.org/10.5061/dryad.0gb5mkm0g
