# Supplementary material for: Population genomics of Mediterranean oat (A. sativa) reveals high genetic diversity and three loci for heading date
Source: Theor Appl Genet. 2021 Mar 26;134(7):2063–77. doi: 10.1007/s00122-021-03805-2 (PMC8263550; doi:10.1007/s00122-021-03805-2)
Supplement: Supplementary file 2 — Supplementary file2 (PDF 2582 kb) [file 122_2021_3805_MOESM2_ESM.pdf]

Theoretical and Applied Genetics

Online Resource

Population genomics of Mediterranean oat (*A. sativa*) reveals high genetic diversity and three loci for heading date.

F. J. Canales[1], G. Montilla-Bascón[1], W. A. Bekele[2], C. Howarth[3], T. Langdon[3], N. Rispail[1], N. Tinker[2], E. Prats[1]

[1]*CSIC, Institute for Sustainable Agriculture, Córdoba, Spain*

[2]*Ottawa Research and Development Centre, Agriculture and Agri-Food Canada, Ottawa, ON, Canada*

[3]*Institute of Biological, Environmental and Rural Sciences, Aberystwyth Univ., Aberystwyth, UK*

For correspondence: Elena Prats

E-mail: elena.prats@ias.csic.es

Online Resource 1. Heading date of the oat collection.

Sample	Co17 Rep1	Co17 Rep2	Co17 Rep3	Co18 Rep1	Co18 Rep2	Co18 Rep3	Sa18 Rep1	Sa18 Rep2	Sa18 Rep3	Mean
FM1	172	162	170	189	173	168	164	169	163	170
FM2	163	162	162	178	165	160	152	151	156	161
FM3	147	168	168	189	175	171	161	163	171	168
FM4	143	145	145	157	153	144	139	137	142	145
FM5	145	145	145	161	153	148	139	139	142	146
FM6	139	147	145	154	152	144	138	136	141	144
FM7	139	145	145	161	152	143	141	141	142	145
FM8	163	172	167	184	178	166	160	162	165	169
FM9	141	145	145	154	152	139	140	138	141	144
FM10	151	153	153	164	163	151	142	139	140	151
FM11	141	142	150	170	156	150	142	141	140	148
FM12	160	163	166	188	183	162	160	162	164	168
FM13	153	152	153	164	155	144	142	146	145	150
FM14	153	156	163	182	176	159	157	155	156	162
FM15	139	143	145	158	151	144	139	138	142	144
FM16	139	145	149	162	152	144	139	141	142	146
FM17	149	157	162	175	167	175	152	152	153	160
FM18	160	163	163	191	174	NA	156	159	161	166
FM19	141	145	145	162	149	144	140	139	142	145
FM20	155	157	153	178	166	166	144	154	153	158
FM21	145	147	152	158	151	147	140	141	142	147
FM22	177	174	181	196	184	174	167	169	169	177
FM23	145	145	143	159	154	144	138	141	140	145
FM24	148	145	141	164	153	152	142	141	144	148
FM25	139	139	135	158	150	138	136	134	139	141
FM26	148	145	145	163	162	151	141	140	142	149
FM27	163	162	166	182	162	166	161	167	155	165
FM28	148	139	145	159	165	146	142	144	143	148
FM29	139	139	139	156	149	139	137	134	137	141

FM30	148	145	153	163	166	151	142	141	145	150
FM31	151	153	153	165	166	154	142	142	151	153
FM32	137	142	141	154	152	140	138	135	139	142
FM33	142	145	145	157	154	144	140	137	142	145
FM34	145	142	145	154	153	140	139	140	142	144
FM35	160	153	153	184	177	158	154	152	155	161
FM36	171	177	170	189	178	171	158	162	174	172
FM37	152	152	153	165	165	154	153	153	154	156
FM38	145	139	141	156	150	144	139	141	141	144
FM39	154	165	166	173	166	155	143	142	145	157
FM40	171	168	171	186	191	176	161	164	171	173
FM41	142	141	145	157	154	144	139	141	142	145
FM42	171	163	168	188	181	166	160	169	167	170
FM43	163	157	172	191	180	173	164	169	172	171
FM44	142	151	150	173	152	149	140	145	143	150
FM45	145	147	147	166	152	152	143	144	144	149
FM46	145	143	145	156	154	141	135	137	141	144
FM47	155	155	170	177	166	166	153	161	156	162
FM48	163	166	174	190	183	169	167	173	171	173
FM49	153	153	163	175	174	143	151	155	155	158
FM50	145	142	145	162	158	152	141	145	142	148
FM51	171	163	171	174	180	169	158	160	161	167
FM52	160	160	160	175	174	156	154	155	144	160
FM53	166	156	163	174	178	160	156	153	156	162
FM54	157	156	160	188	170	163	156	155	156	162
FM55	138	142	150	157	153	143	138	138	140	144
FM56	141	142	145	155	151	144	139	137	139	144
FM57	141	139	139	155	146	144	138	136	138	142
FM58	148	147	148	158	153	145	142	140	142	147
FM59	136	141	145	156	151	146	138	138	138	143
FM60	170	188	167	184	176	173	165	162	163	172

FM61	186	185	168	189	181	171	166	164	171	176
FM62	170	171	163	189	168	171	165	162	165	169
FM63	168	170	168	188	180	169	164	164	165	171
FM64	158	157	162	188	168	162	154	156	158	163
FM65	145	143	150	171	156	154	142	143	145	150
FM66	176	171	177	189	186	171	167	164	164	174
FM67	150	145	NA	163	165	153	142	141	142	150
FM68	148	145	148	171	164	146	143	142	144	150
FM69	160	160	158	184	168	162	154	153	156	162
FM70	162	168	172	187	177	166	165	162	167	170
FM71	178	181	172	185	180	169	160	166	165	173
FM72	145	152	152	171	154	153	141	142	144	150
FM73	153	155	178	171	157	155	145	153	152	158
FM74	145	147	145	159	149	142	139	142	142	146
FM75	163	171	168	191	184	173	164	170	168	173
FM76	141	139	139	162	158	145	140	142	140	145
FM77	157	156	171	175	170	157	155	155	156	161
FM78	153	151	160	175	170	158	144	152	154	158
FM79	152	145	149	158	NA	146	142	141	144	147
FM80	148	145	145	165	156	144	137	141	140	147
FM81	163	167	178	188	174	169	158	138	158	166
FM82	164	163	162	193	182	174	156	158	161	168
FM83	160	160	170	189	180	173	164	175	164	171
FM84	145	143	145	156	151	144	137	138	140	144
FM85	153	153	153	187	166	157	145	157	154	158
FM86	141	141	145	158	150	142	139	140	142	144
FM87	153	153	153	176	166	156	143	143	152	155
FM88	145	145	153	162	153	144	141	139	142	147
FM89	147	145	151	161	153	146	140	140	142	147
FM90	131	141	139	158	146	145	134	138	137	141
FM91	139	145	141	159	151	141	136	138	139	143

FM92	153	155	160	176	166	168	153	152	156	160
FM93	139	141	145	157	146	140	137	137	137	142
FM94	145	143	145	159	147	142	135	133	137	143
FM95	145	145	153	172	163	156	142	143	145	152
FM96	148	145	157	173	162	155	143	144	144	152
FM97	139	141	145	159	151	144	137	137	136	143
FM98	153	153	153	175	166	155	153	154	155	158
FM99	139	142	145	158	153	144	140	141	141	145
FM100	145	145	145	166	154	147	141	141	142	147
FM101	172	185	179	191	183	176	167	173	171	177
FM102	152	153	179	164	168	164	143	153	156	159
FM103	145	142	145	160	154	144	138	140	139	145
FM104	NA	179	181	191	184	171	168	174	137	173
FM105	155	153	160	172	175	157	155	157	154	160
FM106	168	160	170	197	181	169	168	173	180	174
FM107	168	162	162	190	181	169	168	177	165	171
FM108	167	162	163	191	179	169	168	165	165	170
FM109	137	145	145	161	153	155	140	143	142	147
FM110	145	145	152	169	157	154	142	144	143	150
FM111	153	153	154	184	165	158	144	153	156	158
FM112	144	142	145	160	153	142	141	139	142	145
FM113	165	156	166	183	180	165	155	154	156	164
FM114	160	155	153	173	166	157	154	152	153	158
FM115	171	179	168	187	178	163	156	159	157	169
FM116	153	156	153	174	166	162	145	145	152	156
FM117	153	153	154	174	166	162	146	145	150	156
FM118	151	153	153	172	166	164	153	155	152	158
FM119	145	148	150	171	161	149	143	145	144	151
FM120	145	145	145	163	152	143	140	141	141	146
FM121	145	145	147	161	153	144	139	138	142	146
FM122	139	133	141	160	154	146	142	141	143	144

FM123	141	135	145	158	149	144	139	138	139	143
FM124	139	139	139	155	149	138	136	134	137	141
FM125	147	153	155	174	165	155	142	145	143	153
FM126	148	153	153	174	165	155	143	145	144	153
FM127	157	158	160	177	166	158	144	150	146	157
FM128	153	145	153	164	157	156	141	150	142	151
FM129	149	NA	NA	165	154	155	142	150	143	151
FM130	151	145	139	164	166	155	142	145	142	150
FM131	150	NA	NA	165	156	150	142	145	138	150
FM132	152	145	147	160	157	152	142	143	142	149
FM133	153	158	156	175	177	164	154	154	155	161
FM134	152	141	145	164	154	146	141	141	142	147
FM135	153	145	151	166	162	152	144	143	144	151
FM136	156	153	153	173	173	156	145	154	154	157
FM137	152	151	153	176	166	163	143	152	154	157
FM138	142	152	147	161	153	145	138	140	142	147
FM139	145	142	145	159	153	143	144	144	145	147
FM140	156	153	153	189	166	157	156	155	156	160
FM141	160	163	160	186	167	155	156	154	155	162
FM142	139	141	141	161	153	147	141	140	141	145
FM143	184	195	181	201	185	171	171	171	169	181
FM144	160	163	160	182	166	159	156	153	155	162
FM145	163	159	158	177	166	156	154	154	156	160
FM146	151	145	150	165	155	144	140	141	142	148
FM147	170	162	168	194	176	165	156	158	157	167
FM148	157	155	153	174	157	155	144	142	155	155
FM149	153	145	151	165	157	146	142	142	146	150
FM150	153	145	148	168	147	146	142	141	145	148
FM151	163	162	162	190	182	163	157	157	157	166
FM152	NA	181	171	191	181	170	169	164	163	174
FM153	156	178	160	186	166	164	156	162	157	165

FM154	156	163	160	179	167	162	156	156	156	162
FM155	147	145	148	162	154	147	141	144	142	148
FM156	171	174	179	190	180	172	168	170	174	175
FM157	180	177	179	191	183	174	167	168	140	173
FM158	158	159	168	187	177	166	156	159	156	165
FM159	172	160	170	185	183	167	164	162	140	167
FM160	155	162	162	175	181	167	158	155	154	163
FM161	178	174	172	194	184	177	167	162	174	176
FM162	160	170	166	193	181	171	158	159	165	169
FM163	172	172	171	188	180	172	164	162	163	172
FM164	160	170	166	182	173	159	156	156	158	164
FM165	171	181	163	183	178	164	156	157	158	168
FM166	170	171	168	182	183	169	156	157	163	169
FM167	171	163	168	186	173	171	156	156	157	167
FM168	140	145	145	157	147	144	135	135	137	143
FM169	170	160	158	183	168	162	154	158	154	163
FM170	145	145	142	157	146	142	141	138	138	144
FM171	153	151	153	173	156	153	153	143	153	154
FM172	152	145	153	163	165	156	144	142	144	151
FM173	156	142	150	170	157	154	141	141	142	150
FM174	151	145	145	161	152	147	140	137	142	147
FM175	145	145	145	161	154	147	139	141	142	147
FM176	148	145	151	170	165	156	137	143	142	151
FM177	171	172	166	190	181	167	158	157	163	169
FM178	139	142	142	155	154	139	139	139	139	143
FM179	140	142	140	155	148	144	136	139	138	142
FM180	163	183	163	196	170	159	157	162	161	168
FM181	149	145	145	161	151	140	138	141	139	146
FM182	171	153	160	188	167	170	156	160	139	163
FM183	175	160	163	190	180	166	166	167	140	167
FM184	158	153	153	193	169	169	155	160	163	164

FM185	168	166	160	187	171	165	155	162	164	166
FM186	168	157	168	187	174	164	156	155	161	166
FM187	170	163	168	194	181	168	157	162	165	170
FM188	163	155	162	190	183	164	156	157	163	166
FM189	168	162	171	191	181	169	160	157	163	169
FM190	163	172	171	192	169	168	158	162	163	169
FM191	185	172	168	187	167	169	158	162	160	170
FM192	159	160	160	184	180	167	156	162	163	166
FM193	170	181	174	189	180	171	164	164	163	173
FM194	181	168	171	187	180	171	157	157	160	170
FM195	179	195	172	189	182	171	163	162	163	175
FM196	163	163	163	183	178	165	156	160	159	166
FM197	168	171	160	193	166	168	158	155	157	166
FM198	139	135	139	154	142	135	134	133	138	139
FM199	148	145	153	173	153	153	142	142	144	150
FM200	142	139	145	157	143	144	136	137	140	143
FM201	145	145	150	161	153	144	141	139	141	147
FM202	160	155	155	174	166	159	154	152	155	159
FM203	145	140	145	167	156	141	137	140	142	146
FM204	141	145	145	158	162	145	140	142	144	147
FM205	142	141	145	158	156	146	142	138	142	146
FM206	139	141	142	155	146	144	137	138	137	142
FM207	136	141	139	151	140	137	134	141	135	139
FM208	160	163	162	186	169	159	155	154	154	163
FM209	153	145	142	166	152	144	141	143	140	147
FM210	145	145	148	160	151	142	138	141	138	145
FM211	157	147	145	175	158	156	144	152	146	153
FM212	160	163	160	186	174	159	158	161	157	164
FM213	162	162	162	194	181	171	158	155	157	167
FM214	179	145	181	193	184	176	167	138	138	167
FM215	160	162	168	191	181	169	156	156	161	167

FM216	168	167	168	191	178	168	160	162	165	170
FM217	179	171	171	193	184	171	165	164	164	173
FM218	160	157	160	185	175	162	156	159	160	164
FM219	186	184	181	186	180	171	164	166	172	177
FM220	186	184	181	189	184	171	165	164	165	176
FM221	176	162	168	189	182	174	166	165	176	173
FM222	178	195	181	189	182	171	168	170	166	178
FM223	168	163	166	187	176	167	156	157	158	166
FM224	176	181	153	163	149	144	142	141	142	155
FM225	171	171	170	182	175	164	161	162	163	169
FM226	168	168	178	191	182	171	156	153	164	170
FM227	171	163	183	198	182	NA	166	163	163	174
FM228	168	170	172	189	177	170	171	163	165	172
FM229	174	181	179	192	182	171	165	164	163	174
FM230	179	181	175	189	182	173	168	164	165	175
FM231	191	181	181	191	NA	178	167	166	169	178
FM232	153	153	153	172	163	152	146	145	144	154
FM233	139	141	139	157	142	142	135	139	136	141
FM234	174	171	168	185	168	162	160	163	157	168
FM235	156	163	153	177	170	163	159	162	140	160
FM236	152	145	156	166	157	151	145	153	146	152
FM237	153	148	152	165	152	149	139	144	139	149
FM238	142	135	145	158	154	146	156	160	161	151
FM239	160	157	160	175	167	166	156	158	156	162
FM240	153	152	155	178	169	156	156	154	155	159
FM241	172	170	172	191	181	169	159	164	165	171
FM242	148	145	163	174	164	149	142	143	144	153
FM243	171	174	172	194	183	169	161	167	168	173
FM244	145	145	145	164	154	149	137	141	142	147
FM245	153	157	159	175	166	155	137	141	144	154
FM246	143	143	142	171	157	146	138	141	142	147

FM247	171	153	158	174	180	155	142	143	146	158
FM248	138	136	139	159	146	143	134	134	136	140
FM249	162	158	163	184	178	157	156	155	155	163
FM250	160	160	163	178	166	167	154	155	156	162
FM251	163	162	168	192	167	168	156	155	156	165
FM252	168	166	168	186	150	164	158	159	158	164
FM253	150	148	145	170	153	153	153	141	144	151
FM254	163	163	163	191	177	171	152	153	159	166
FM255	155	157	153	193	178	159	155	151	154	162
FM256	157	156	163	193	170	154	151	155	156	162
FM257	156	158	155	183	178	158	154	152	152	161
FM258	158	160	163	188	174	166	151	155	155	163
FM259	156	160	166	177	174	155	151	152	154	161
FM260	148	153	153	174	157	148	142	144	143	151
FM261	147	145	145	170	157	148	144	146	137	149
FM262	149	148	153	173	158	155	142	145	143	152
FM263	153	145	157	186	157	164	143	153	146	156
FM264	145	136	148	174	154	152	142	143	142	148
FM265	147	139	152	171	158	147	142	143	144	149
FM266	142	138	145	166	158	154	139	143	142	148
FM267	153	155	152	189	178	160	151	154	151	160
FM268	153	151	147	175	159	154	144	143	146	153
FM269	151	148	153	178	157	152	143	144	146	152
FM270	144	136	139	165	146	138	136	139	140	143
FM271	148	148	147	164	165	152	142	145	143	150
FM272	140	143	145	163	163	147	139	141	142	147
FM273	145	151	149	170	156	153	142	142	143	150
FM274	151	151	145	165	165	154	142	143	146	151
FM275	145	145	145	171	155	156	143	143	146	150
FM276	153	153	145	164	157	148	145	144	146	151
FM277	147	148	145	171	171	156	142	143	145	152

FM278	150	153	147	172	155	156	143	142	145	151
FM279	146	153	148	171	153	156	146	143	145	151
FM280	150	152	153	171	163	152	144	143	144	152
FM281	135	131	133	155	139	140	134	133	132	137
FM282	156	145	145	165	155	154	142	140	142	149
FM283	147	145	150	161	153	148	142	141	142	148
FM284	163	160	168	191	187	174	167	167	164	171
FM285	153	153	165	179	177	156	146	151	151	159
FM286	153	153	156	187	166	155	156	153	152	159
FM287	157	156	160	187	179	165	167	164	139	164
FM288	163	163	172	194	184	169	167	166	166	171
FM289	162	160	178	196	183	171	170	169	167	173
FM290	153	153	160	177	168	157	154	155	154	159
FM291	156	163	162	194	181	165	168	167	161	168
FM292	156	153	155	194	178	159	156	159	156	163
FM293	162	162	165	194	176	163	160	168	161	168
FM294	160	160	163	191	184	163	164	161	163	168
FM295	156	155	153	186	170	156	156	156	156	161
FM296	156	153	153	190	168	159	159	157	156	161
FM297	153	153	153	187	166	158	156	154	155	160
FM298	153	158	158	185	166	157	156	155	156	160
FM299	153	153	153	176	166	158	156	155	157	158
FM300	160	162	159	182	165	155	155	154	155	161
FM301	166	178	174	193	186	172	168	170	168	175
FM302	155	153	155	177	166	156	156	155	156	159
FM303	166	170	181	189	180	167	156	156	158	169
FM304	163	168	163	182	170	158	156	155	156	164
FM305	153	158	153	174	165	154	144	150	152	156
FM306	NA	175	170	189	178	167	167	162	159	171
FM307	162	162	179	NA	180	172	168	161	168	169
FM308	168	162	160	190	169	169	166	162	163	168

FM309	168	163	162	192	183	171	166	161	165	170
FM310	163	163	162	193	166	157	166	157	158	165
FM311	170	176	174	191	181	173	165	167	162	173
FM312	160	171	171	191	183	172	158	162	160	170
FM313	181	155	181	191	183	NA	146	145	138	165
FM314	157	155	155	184	170	156	146	150	153	158
FM315	153	153	160	195	184	166	153	156	139	162
FM316	149	153	157	175	162	150	152	152	145	155
FM317	149	153	155	175	166	154	152	151	146	156
FM318	152	156	153	187	165	159	153	153	149	159
FM319	163	160	163	195	183	167	167	168	163	170
FM320	160	160	167	194	181	168	164	164	162	169
FM321	160	163	165	195	185	168	169	168	163	171
FM322	148	148	152	190	169	158	156	157	156	159
FM323	163	163	168	194	180	169	168	165	163	170
FM324	149	153	148	187	166	155	158	154	156	159
FM325	171	163	171	192	184	171	164	173	163	172
FM326	156	160	160	184	179	171	163	162	163	166
FM327	156	162	162	191	176	170	164	162	161	167
FM328	155	156	155	186	180	168	158	161	158	164
FM329	159	157	156	177	166	169	146	148	146	158
FM330	153	157	153	182	169	158	156	157	159	161
FM331	NA	NA	165	171	163	152	142	143	144	154
FM332	156	171	168	191	180	171	161	162	163	169
FM333	168	168	158	191	178	167	160	159	163	168
FM334	168	179	180	191	180	171	164	168	171	175
FM335	166	168	168	185	169	165	164	162	162	168
FM336	NA	171	171	191	182	169	163	164	164	172
FM337	159	160	160	175	166	156	153	153	151	159
FM338	153	153	153	174	166	156	146	145	153	155
FM339	156	153	153	175	166	154	146	150	138	155

FM340	180	160	NA	195	186	179	155	156	153	171
FM341	171	181	174	185	181	170	160	165	165	172
FM342	172	178	175	195	185	172	168	166	163	175
FM343	153	153	166	187	178	164	156	164	157	164
FM344	162	162	171	191	181	170	169	166	165	171
FM345	160	153	160	186	169	163	168	162	158	164
FM346	163	158	163	195	183	171	158	173	163	170
FM347	151	145	148	172	155	147	141	144	142	150
FM348	156	155	157	166	165	154	145	151	144	155
FM349	157	156	156	184	174	158	156	155	156	161
FM350	153	148	167	187	169	164	157	156	156	162
FM351	153	158	172	186	177	165	160	158	159	165
FM352	181	195	NA	NA	187	173	169	169	163	177
FM353	170	175	168	186	178	169	158	158	162	169
FM354	181	181	NA	192	184	175	164	164	166	176
FM355	170	171	172	189	184	172	158	162	160	171
FM356	181	181	181	191	186	170	164	164	166	176
FM357	162	167	160	181	166	165	154	155	155	163
FM358	145	148	160	172	156	146	135	140	138	149
FM359	153	156	150	176	165	154	142	143	142	153
FM360	151	148	153	173	155	154	136	141	138	150
FM361	151	153	148	164	153	146	134	138	141	148
FM362	151	148	155	174	155	148	154	152	146	154
FM363	145	148	145	165	165	146	136	140	142	148
FM364	155	157	157	176	180	158	154	157	144	160
FM365	147	148	152	174	166	145	142	139	136	150
FM366	151	150	150	173	166	156	134	141	142	152
FM367	153	153	156	184	174	159	155	155	155	161
FM368	143	145	141	159	153	144	139	139	142	145
FM369	145	140	140	160	152	144	140	141	140	145
FM370	153	153	157	177	176	173	144	155	144	159

FM371	163	147	151	174	165	147	146	159	152	156
FM372	162	157	163	184	174	164	156	155	154	163
FM373	145	142	145	162	153	138	139	141	142	145
FM374	166	160	162	185	170	159	156	156	156	163
FM375	160	167	160	184	176	162	155	153	155	164
FM376	153	152	160	184	166	162	153	152	153	159
FM377	155	153	160	177	163	155	146	150	151	157
FM378	150	151	149	160	164	155	138	134	142	149
FM379	148	152	148	174	158	157	146	148	145	153
FM380	141	141	145	161	151	147	136	142	142	145
FM381	153	153	153	182	170	160	154	156	155	160
FM382	145	145	148	174	157	146	142	145	143	149
FM383	142	142	139	158	151	147	138	143	140	144
FM384	140	139	139	158	154	145	140	144	140	144
FM385	160	153	160	182	180	161	154	158	153	162
FM386	147	145	149	163	156	149	142	146	143	149
FM387	156	153	153	174	168	161	151	154	144	157
FM388	155	158	153	176	169	160	152	157	153	159
FM389	153	160	153	176	168	161	155	155	155	160
FM390	151	153	153	182	169	159	153	155	154	159
FM391	153	153	153	185	168	156	154	155	139	157
FM392	150	151	152	176	165	150	143	146	146	153
FM393	145	153	145	170	166	149	143	144	145	151
FM394	153	153	153	171	167	157	154	152	152	157
FM395	155	153	156	170	155	157	155	155	152	156
FM396	146	145	150	170	166	157	143	143	142	151
FM397	153	156	157	170	167	159	152	154	152	158
FM398	153	153	160	190	171	169	155	157	154	162
FM399	160	157	160	179	169	168	157	159	156	163
FM400	160	157	157	181	178	168	158	164	157	164
FM401	147	133	139	166	155	147	139	143	140	145

FM402	153	153	150	170	164	156	145	154	146	155
FM403	145	145	151	157	153	143	140	143	142	147
FM404	155	152	156	170	169	159	147	154	153	157
FM405	148	150	152	167	155	154	145	143	143	151
FM406	126	133	133	157	148	145	138	144	140	140
FM407	141	139	140	157	156	145	139	144	142	145
FM408	158	153	NA	175	182	164	155	158	154	162
FM409	156	157	165	183	179	161	153	156	152	163
FM410	151	153	153	174	167	161	145	155	152	157
FM411	163	162	160	184	173	166	156	160	156	165
FM412	NA	168	NA	186	179	161	156	161	156	167
FM413	156	160	155	187	171	166	155	158	155	163
FM414	160	162	157	182	170	164	155	157	154	162
FM415	160	160	NA	176	176	166	156	156	156	163
FM416	153	162	156	177	167	157	154	154	140	158
FM417	156	157	158	187	181	159	156	158	138	161
FM418	141	145	145	161	154	145	140	144	141	146
FM419	139	145	142	161	155	146	142	140	140	146
FM420	153	158	153	173	175	161	154	156	154	160
FM421	153	153	153	173	171	157	153	154	151	158
FM422	152	153	145	169	157	147	143	145	143	151
FM423	153	155	153	173	170	157	147	155	152	157
FM424	153	157	153	173	177	160	146	155	154	159
FM425	153	151	153	173	167	160	154	156	154	158
FM426	153	155	153	178	169	154	154	156	153	158
FM427	157	153	157	171	168	159	147	154	153	158
FM428	153	153	155	170	167	160	139	155	151	156
FM429	153	148	153	170	168	159	147	154	153	156
FM430	160	155	163	181	169	161	156	157	156	162
FM431	153	153	160	181	168	168	157	157	155	161
FM432	145	142	145	165	155	149	145	143	140	148

FM433	139	137	145	165	156	145	142	146	142	146
FM434	151	148	148	173	170	159	152	156	150	156
FM435	160	150	148	186	172	158	146	161	158	160
FM436	148	153	148	174	168	150	146	147	145	153
FM437	159	158	158	178	170	161	156	158	155	162
FM438	156	157	163	188	167	176	153	158	155	164
FM439	153	156	148	189	168	169	145	147	144	158
FM440	162	162	171	189	179	165	158	158	156	167
FM441	143	148	145	161	156	150	142	144	142	148
FM442	152	153	151	176	160	157	153	155	142	155
FM443	150	151	148	174	165	148	145	147	145	153
FM444	156	158	153	192	178	171	167	158	156	165
FM445	148	145	139	175	156	156	142	155	144	151
FM446	153	161	153	182	176	160	153	153	154	161
FM447	132	138	126	161	152	138	135	139	137	140
FM448	153	145	153	172	167	157	153	155	145	156
FM449	153	155	153	190	169	161	159	163	158	162
FM450	142	152	145	170	155	146	138	143	142	148
FM451	139	141	139	155	155	143	140	143	140	144
FM452	151	152	151	170	157	159	143	156	145	154
FM453	153	153	152	171	179	161	153	157	153	159
FM454	168	180	160	182	184	176	157	161	158	170
FM455	150	150	150	169	167	152	154	157	153	156
FM456	145	143	145	157	155	147	143	143	142	147
FM457	162	163	162	186	186	184	159	173	156	170
FM458	150	147	149	166	167	160	152	158	154	156
FM459	145	147	145	157	155	149	143	143	142	147
FM460	151	153	148	173	166	157	151	155	144	155
FM461	155	155	158	174	169	159	156	158	156	160
FM462	140	141	139	159	153	146	138	144	141	145
FM463	125	131	129	161	148	141	132	138	131	137

FM464	153	153	151	185	165	164	154	158	154	160
FM465	145	141	141	172	156	147	145	147	144	149
FM466	145	143	145	165	159	146	141	143	141	148
FM467	143	143	145	173	164	150	143	144	144	150
FM468	162	NA	160	189	171	168	150	154	154	164
FM469	141	145	139	160	151	148	141	144	142	146
FM470	145	139	139	161	159	143	137	142	139	145
FM471	150	148	148	172	159	155	143	153	144	152
FM472	150	147	148	174	164	154	142	153	146	153
FM473	163	162	160	174	167	157	153	155	152	160
FM474	143	153	145	170	157	147	143	147	144	150
FM475	140	149	139	157	155	144	135	140	139	144
FM476	153	153	148	168	155	154	144	147	146	152
FM477	148	153	148	172	175	156	145	145	145	154
FM478	150	147	145	168	166	159	143	153	145	153
FM479	151	148	150	169	156	159	143	155	146	153
FM480	152	145	148	169	163	149	143	153	144	152
FM481	148	145	145	169	163	150	141	141	144	150
FM482	145	143	145	168	156	148	142	141	144	148
FM483	149	148	145	157	156	150	143	154	145	150
FM484	152	145	148	169	157	159	145	159	143	153
FM485	118	118	118	149	137	133	125	131	126	128
FM486	163	181	162	184	183	NA	161	171	158	170
FM487	157	157	160	187	177	171	168	167	165	168
FM488	168	179	170	193	183	176	171	172	169	176
FM489	163	163	163	188	170	166	166	165	163	168
FM490	160	162	167	192	185	176	170	169	166	172
FM491	149	151	153	178	163	159	147	148	151	155
FM492	159	160	157	189	181	168	160	162	157	166
FM493	154	156	153	183	176	157	156	163	157	162
FM494	155	162	163	182	176	168	156	158	156	164

FM495	153	155	160	185	168	155	156	158	156	161
FM496	148	156	160	176	168	155	156	157	156	159
FM497	153	157	153	186	168	169	157	158	156	162
FM498	157	168	157	194	181	168	135	178	165	167
FM499	157	162	158	194	183	177	161	165	161	169
FM500	157	168	156	192	181	170	161	165	159	168
FM501	163	171	174	200	190	181	163	174	163	176
FM502	163	181	174	186	187	182	164	168	167	175
FM503	163	168	160	185	167	162	160	169	159	166
FM504	157	160	153	186	NA	159	154	157	154	160
FM505	153	147	151	171	166	159	146	160	153	156
FM506	153	148	148	183	168	161	157	164	156	160
FM507	153	148	150	182	169	161	157	161	156	160
FM508	165	171	160	186	177	159	155	157	154	165
FM509	149	144	145	170	167	156	145	147	152	153
FM510	163	147	151	168	169	149	146	157	146	155
FM511	160	153	160	186	170	171	157	175	156	165
FM512	160	153	155	186	171	159	159	160	158	162
FM513	157	187	160	184	179	168	161	162	160	169
FM514	156	157	160	189	180	169	160	166	156	166
FM515	159	155	158	176	168	157	155	158	154	160
FM516	149	153	151	173	164	151	153	153	153	155
FM517	139	140	141	163	156	145	139	142	142	145
FM518	153	153	150	165	164	147	144	145	143	152
FM519	135	139	135	165	156	144	138	136	140	143
FM520	138	138	139	171	164	160	143	146	145	149
FM521	147	152	145	174	166	149	142	146	144	152
FM522	139	145	139	160	154	145	142	144	142	146
FM523	135	140	139	163	148	141	137	136	136	142
FM524	145	145	148	171	151	147	142	144	141	148
FM525	145	145	153	174	166	150	142	144	142	151

FM526	156	153	160	176	168	167	146	155	152	159
FM527	147	148	152	174	164	152	143	151	144	153
FM528	145	147	145	170	157	150	144	155	142	151
FM529	145	148	145	158	157	142	140	143	142	147
FM530	118	125	118	152	133	138	133	132	126	131
FM531	139	145	135	157	147	147	141	143	142	144
FM532	145	147	145	172	163	150	143	147	142	151
FM533	156	148	153	170	163	150	153	154	144	155
FM534	156	147	150	167	165	145	142	155	145	153
FM535	163	153	160	172	167	160	154	157	154	160
FM536	153	147	150	168	157	156	145	155	146	153
FM537	145	134	138	153	150	145	138	135	135	142
FM538	133	125	118	150	133	136	130	133	129	132
FM539	118	118	118	150	133	136	129	131	125	129
FM540	145	153	145	159	155	147	143	147	142	148
FM541	134	139	136	158	148	140	137	143	134	141
FM542	153	158	157	174	170	159	156	158	154	160
FM543	159	163	160	186	178	161	157	160	156	164
FM544	156	156	156	178	176	157	155	157	153	160
FM545	142	149	142	173	156	149	152	156	151	152
FM546	153	158	163	189	187	171	164	166	163	168
FM547	152	167	158	189	179	167	156	165	143	164
FM548	147	155	153	178	160	149	155	162	153	157
FM549	148	153	148	187	168	171	156	160	156	161
FM550	153	171	155	177	166	157	158	157	155	161
FM551	153	163	156	189	170	169	158	158	156	164
FM552	162	162	168	191	171	171	165	160	162	168
FM553	177	178	181	191	183	172	166	171	163	176
FM554	160	155	151	178	178	159	154	154	154	160
FM555	156	162	160	182	177	171	157	161	155	165
FM556	151	158	150	174	175	159	141	141	139	154

FM557	152	153	149	173	166	160	146	155	146	156
FM558	156	153	145	172	158	161	152	NA	153	156
FM559	151	145	149	166	164	158	143	144	142	151
FM560	163	157	162	181	168	161	157	167	155	164
FM561	153	145	142	171	152	149	142	145	143	149
FM562	156	148	150	161	167	157	144	145	145	153
FM563	155	152	148	170	156	159	146	145	144	153
FM564	156	153	166	186	168	166	147	156	155	161
FM565	159	157	156	172	163	157	156	157	155	159
FM566	142	139	139	153	152	149	143	145	142	145
FM567	139	145	137	160	150	142	140	140	142	144
FM568	147	145	148	161	160	147	142	148	145	149
FM569	147	145	148	163	160	148	142	148	145	150
FM570	148	145	145	170	161	147	144	155	151	152
FM571	149	148	147	164	160	145	142	148	142	149
FM572	149	150	145	165	167	155	144	148	144	152
FM573	151	157	155	178	183	157	151	153	145	159
FM574	148	145	145	173	165	164	144	155	153	155
FM575	160	162	156	182	171	159	155	158	156	162
FM576	153	156	157	185	176	164	154	157	153	162
FM577	153	156	158	174	167	159	152	156	154	159
FM578	157	163	158	184	168	161	156	158	157	163
FM579	155	157	153	176	164	161	155	156	151	159
FM580	157	163	160	178	170	161	159	159	156	163
FM581	153	157	153	178	168	159	156	157	155	160
FM582	153	153	153	169	165	157	155	157	151	157
FM583	160	168	163	175	168	162	157	157	156	163
FM584	157	162	157	175	166	166	155	157	156	161
FM585	145	147	141	169	155	147	145	155	138	149
FM586	158	160	159	179	167	162	155	157	153	161
FM587	162	157	158	180	169	161	156	157	153	162

FM588	162	162	157	173	168	161	157	157	154	161
FM589	153	151	150	160	157	149	145	147	146	151
FM590	153	155	155	170	167	164	146	153	155	158
FM591	156	153	148	170	168	160	147	152	152	156
FM592	145	145	149	168	155	147	144	154	144	150
FM593	157	156	157	174	165	160	156	157	154	160
FM594	153	156	157	168	166	159	154	157	154	158
FM595	179	171	174	187	176	168	158	166	167	172
FM596	162	157	162	184	179	164	155	158	155	164
FM597	145	153	145	164	154	148	144	146	145	149
FM598	155	156	156	173	171	159	155	148	146	158
FM599	148	155	153	176	171	157	145	148	143	155
FM600	139	145	147	171	156	149	142	144	142	148
FM601	156	185	168	177	177	170	158	158	165	168
FM602	153	157	162	183	170	157	156	157	155	161
FM603	140	145	139	170	154	148	133	134	139	145
FM604	153	163	155	185	171	168	145	148	146	159
FM605	151	160	156	185	170	161	146	156	146	159
FM606	155	160	155	186	178	167	152	155	152	162
FM607	133	145	132	159	148	140	137	141	140	142
FM608	153	153	151	171	164	151	145	146	145	153
FM609	163	157	157	173	166	159	147	157	152	159
FM610	149	153	145	166	160	150	146	147	142	151
FM611	160	162	162	180	167	161	155	159	153	162
FM612	151	155	152	159	158	149	144	153	151	153
FM613	145	143	145	159	149	151	135	144	138	146
FM614	157	145	145	170	160	159	138	157	140	152
FM615	145	145	148	166	156	150	142	147	142	149
FM616	148	145	149	167	164	148	143	147	143	150
FM617	152	151	148	167	164	154	144	145	143	152
FM618	156	156	158	179	166	159	157	157	155	160

FM619	155	156	156	170	167	160	157	157	156	159
FM620	153	162	156	172	165	167	157	157	156	161
FM621	160	171	163	179	179	163	158	164	159	166
FM622	153	153	152	174	169	154	154	158	153	158
FM623	153	153	155	173	176	157	154	158	155	159
FM624	139	148	153	171	164	149	143	156	151	153
FM625	150	155	155	170	158	155	147	147	146	154
FM626	152	153	153	172	164	157	144	156	153	156
FM627	160	162	171	182	183	166	156	158	144	165
FM628	150	157	153	174	166	157	155	157	153	158
FM629	157	162	162	184	170	161	156	159	158	163
FM630	160	163	172	188	182	171	174	161	161	170
FM631	155	157	158	179	168	161	156	158	156	161
FM632	156	156	156	182	171	167	156	157	156	162
FM633	156	155	156	177	166	159	154	156	155	159
FM634	153	162	158	175	168	159	154	157	154	160
FM635	147	160	157	177	156	149	142	146	142	153
FM636	151	153	145	177	165	147	143	153	151	154
FM637	142	147	145	159	155	152	143	143	141	148
FM638	145	151	153	175	155	150	146	150	144	152
FM639	153	156	154	167	167	161	146	157	145	156
FM640	145	153	145	170	153	158	141	154	150	152
FM641	142	145	148	152	155	147	137	141	142	145
FM642	145	145	145	170	155	148	142	156	142	150
FM643	118	118	118	143	NA	129	125	126	127	125
FM644	160	163	160	179	166	159	147	156	154	160
FM645	147	145	150	171	156	148	145	145	144	150
FM646	145	145	148	168	155	149	143	144	140	149
FM647	145	151	148	166	155	147	143	152	142	150
FM648	152	155	153	173	165	154	156	157	153	158
FM649	145	150	151	164	158	155	142	148	144	151

FM650	145	147	151	163	160	154	142	148	144	150
FM651	160	153	153	177	171	158	156	158	156	160
FM652	156	162	162	165	160	156	143	144	153	156
FM653	NA	195	171	186	183	166	160	163	165	174
FM654	156	156	155	178	169	157	154	157	156	160
FM655	149	149	153	171	168	149	143	146	153	153
FM656	150	153	159	183	168	NA	145	155	144	157
FM657	153	149	156	176	164	156	143	145	143	154
FM658	142	141	133	171	156	147	144	143	142	147
FM659	156	162	156	178	169	155	156	157	155	161
FM660	151	157	153	176	167	150	146	155	153	157
FM661	153	163	160	174	168	155	146	155	152	159
FM662	147	158	151	174	167	177	145	154	145	158
FM663	153	153	145	168	167	157	143	154	144	154
FM664	153	155	155	181	169	159	157	157	156	160
FM665	140	141	145	158	155	146	141	143	142	146
FM666	153	160	171	171	177	167	146	157	152	162
FM667	136	133	131	157	148	135	136	143	140	140
FM668	118	125	118	154	145	135	127	134	134	132
FM669	148	152	145	172	167	160	156	157	156	157
FM670	162	155	160	186	181	168	157	160	163	166
FM671	133	133	125	144	141	130	131	131	133	133
FM672	153	145	151	171	167	159	156	157	154	157
FM673	160	163	163	182	167	160	155	162	156	163
FM674	118	118	118	155	133	131	125	136	127	129
FM675	141	145	139	167	154	145	140	145	141	146
FM676	153	145	145	174	156	157	141	144	142	151
FM677	167	168	166	178	173	167	156	166	142	165
FM678	162	160	160	178	171	160	160	164	156	163
FM679	151	153	153	161	168	157	154	146	154	155
FM680	142	145	141	156	153	142	141	144	142	145

FM681	145	145	145	171	165	145	142	144	144	150
FM682	160	160	160	186	179	160	158	158	156	164
FM683	168	181	168	189	182	171	161	162	161	171
FM684	126	134	133	161	141	145	134	132	138	138
FM685	153	153	148	187	167	158	156	155	154	159
FM686	153	153	150	189	182	159	155	155	154	161
FM687	145	148	147	188	166	158	154	147	153	156
FM688	118	133	118	158	136	145	132	128	130	133
FM689	156	160	162	192	179	159	157	157	156	164
FM690	153	157	153	184	167	158	155	157	155	160
FM691	139	139	149	157	148	144	138	136	137	143
FM692	139	139	150	150	151	159	135	135	140	144
FM693	145	145	139	158	155	149	138	142	139	146
FM694	151	139	139	157	140	142	135	143	142	143
FM695	141	136	133	147	148	144	133	135	136	139
FM696	160	159	160	182	168	159	157	161	156	162
FM697	147	145	151	183	169	147	155	156	154	156
FM698	160	160	160	174	167	159	156	157	156	161
FM699	143	142	141	155	155	148	143	143	142	146
FM700	142	153	148	157	164	158	137	142	142	149
FM701	136	134	132	157	139	145	135	138	139	140
FM702	141	140	139	152	153	148	140	141	149	145
FM703	147	148	151	169	184	155	145	154	153	156
FM704	118	118	118	158	140	138	134	129	132	132
FM705	150	150	148	174	164	151	149	151	149	154
FM706	160	163	167	189	183	171	156	158	163	168
FM707	148	153	155	187	180	157	155	148	156	160
FM708	145	141	147	161	167	145	142	144	142	148
FM709	155	155	156	192	168	171	156	160	158	164

Online Resource 2 Working correspondence between Mrg consensus groups and chromosome identities in the above diploid, *A. atlantica* and *A. eriantha* chromosomes, and hexaploid pseudomolecule based on the best match of ten random markers for each of the 21 consensus linkages groups reported for *A. sativa* (Bekele et al., 2018).

**A. Sativa consensus (Mrg) linkage group**	**Hexaploid oat reference OT3098 v1 chromosomes**	***A. atlantica /A. eriantha* chromosomes**
1	1D	AA2/AA6
2	7D	AA1
3	5C	AE1/AE4/AE6
4	6D	AA7/AA5
5	6A	AA7/AA5
6	5D	AE4/AA4/AA6
8	2D	AA5/AE6
9	4C	AE2/AE1
11	7C	AE7/AE3
12	7A	AA1
13	2C	AE4/AE5/AE7
15	3C	AE3/AE7
17	6C	AE2/AE6
18	1A	AE7/AA2
19	3D	AE5
20	4A and 4D	AA4/AA7
21	4D and 4A	AA4/AA1/AA7/AE7
23	3A	AA3/AA5
24	5A and 5D	AA6/AA4
28	1C	AA3/AA2/AE5
33	2A	AA5

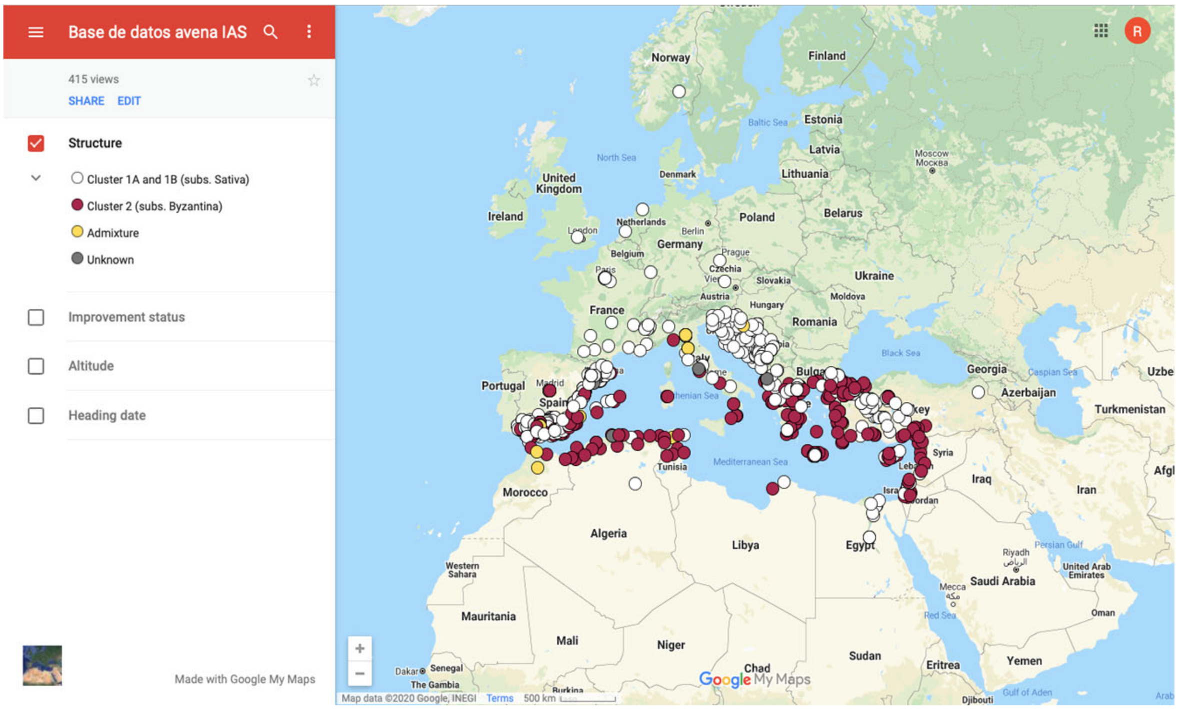

Online Resource 3 Example of the information displayed by the interactive map for each accession comprising data of country of origin, subspecies, collecting date and place (with the corresponding longitude and altitude), type of material as well of detailed pictures of the spike morphology and seed morphology and color. Details on the map functioning can be accessed through the uploaded video

Online Resource 5. Key file for GBS pipeline

Flowcell	lane	Barcode	sample	Plate	Row	Column	LineID
HI.4765.001.Oat-Plate1	1	CTCG	FM710	1	A	1	Blank
HI.4765.001.Oat-Plate1	1	AGCG	FM1	1	A	2	M-001
HI.4765.001.Oat-Plate1	1	TTCTG	FM2	1	A	3	M-002
HI.4765.001.Oat-Plate1	1	ATTGA	FM3	1	A	4	M-003
HI.4765.001.Oat-Plate1	1	TCGTT	FM4	1	A	5	M-004
HI.4765.001.Oat-Plate1	1	GAGATA	FM5	1	A	6	M-005
HI.4765.001.Oat-Plate1	1	CTATTA	FM6	1	A	7	M-006
HI.4765.001.Oat-Plate1	1	CTTGCTT	FM7	1	A	8	M-007
HI.4765.001.Oat-Plate1	1	AATATGG	FM8	1	A	9	M-008
HI.4765.001.Oat-Plate1	1	GCGGAAT	FM9	1	A	10	M-009
HI.4765.001.Oat-Plate1	1	TGCAAGGA	FM10	1	A	11	M-010
HI.4765.001.Oat-Plate1	1	CCATGGGT	FM11	1	A	12	M-011
HI.4765.001.Oat-Plate1	1	TGCA	FM12	1	B	1	M-012
HI.4765.001.Oat-Plate1	1	GATG	FM13	1	B	2	M-013
HI.4765.001.Oat-Plate1	1	AGCCG	FM14	1	B	3	M-014
HI.4765.001.Oat-Plate1	1	CATCT	FM15	1	B	4	M-015
HI.4765.001.Oat-Plate1	1	GGTTGT	FM16	1	B	5	M-016
HI.4765.001.Oat-Plate1	1	ATGCCT	FM17	1	B	6	M-017
HI.4765.001.Oat-Plate1	1	GCCAGT	FM18	1	B	7	M-018
HI.4765.001.Oat-Plate1	1	ATGAAAG	FM19	1	B	8	M-019
HI.4765.001.Oat-Plate1	1	ACGTGTT	FM20	1	B	9	M-020
HI.4765.001.Oat-Plate1	1	TAGCGGA	FM21	1	B	10	M-021
HI.4765.001.Oat-Plate1	1	TGGTACGT	FM22	1	B	11	M-022
HI.4765.001.Oat-Plate1	1	CGCGGAGA	FM23	1	B	12	M-023
HI.4765.001.Oat-Plate1	1	ACTA	FM24	1	C	1	M-024
HI.4765.001.Oat-Plate1	1	TCAG	FM25	1	C	2	M-025
HI.4765.001.Oat-Plate1	1	GTATT	FM26	1	C	3	M-026
HI.4765.001.Oat-Plate1	1	CCTAG	FM27	1	C	4	M-027
HI.4765.001.Oat-Plate1	1	CCACGT	FM28	1	C	5	M-028
HI.4765.001.Oat-Plate1	1	AGTGGA	FM29	1	C	6	M-029
HI.4765.001.Oat-Plate1	1	GGAAGA	FM30	1	C	7	M-030
HI.4765.001.Oat-Plate1	1	AAAAGTT	FM31	1	C	8	M-031
HI.4765.001.Oat-Plate1	1	ATTAATT	FM32	1	C	9	M-032
HI.4765.001.Oat-Plate1	1	TCGAAGA	FM33	1	C	10	M-033
HI.4765.001.Oat-Plate1	1	TCTCAGTG	FM34	1	C	11	M-034
HI.4765.001.Oat-Plate1	1	CGTGTGGT	FM35	1	C	12	M-035
HI.4765.001.Oat-Plate1	1	CAGA	FM36	1	D	1	M-036
HI.4765.001.Oat-Plate1	1	TGCGA	FM37	1	D	2	M-037
HI.4765.001.Oat-Plate1	1	CTGTA	FM38	1	D	3	M-038
HI.4765.001.Oat-Plate1	1	GAGGA	FM39	1	D	4	M-039
HI.4765.001.Oat-Plate1	1	TTCAGA	FM40	1	D	5	M-040
HI.4765.001.Oat-Plate1	1	ACCTAA	FM41	1	D	6	M-041
HI.4765.001.Oat-Plate1	1	GTACTT	FM42	1	D	7	M-042
HI.4765.001.Oat-Plate1	1	GAATTCA	FM43	1	D	8	M-043
HI.4765.001.Oat-Plate1	1	ATTGGAT	FM44	1	D	9	M-044
HI.4765.001.Oat-Plate1	1	TCTGTGA	FM45	1	D	10	M-045
HI.4765.001.Oat-Plate1	1	CGCGATAT	FM46	1	D	11	M-046
HI.4765.001.Oat-Plate1	1	GCTGTGGA	FM47	1	D	12	M-047

HI.4765.001.Oat-Plate1	1	AACT	FM48	1	E	1	M-048
HI.4765.001.Oat-Plate1	1	CGCTT	FM49	1	E	2	M-049
HI.4765.001.Oat-Plate1	1	ACCGT	FM50	1	E	3	M-050
HI.4765.001.Oat-Plate1	1	GGAAG	FM51	1	E	4	M-051
HI.4765.001.Oat-Plate1	1	TAGGAA	FM52	1	E	5	M-052
HI.4765.001.Oat-Plate1	1	ATATGT	FM53	1	E	6	M-053
HI.4765.001.Oat-Plate1	1	GTTGAA	FM54	1	E	7	M-054
HI.4765.001.Oat-Plate1	1	GAACTTG	FM55	1	E	8	M-055
HI.4765.001.Oat-Plate1	1	CATAAGT	FM56	1	E	9	M-056
HI.4765.001.Oat-Plate1	1	TGCTGGA	FM57	1	E	10	M-057
HI.4765.001.Oat-Plate1	1	CGCCTTAT	FM58	1	E	11	M-058
HI.4765.001.Oat-Plate1	1	GGATTGGT	FM59	1	E	12	M-059
HI.4765.001.Oat-Plate1	1	GCGT	FM60	1	F	1	M-060
HI.4765.001.Oat-Plate1	1	TCACG	FM61	1	F	2	M-061
HI.4765.001.Oat-Plate1	1	GCTTA	FM62	1	F	3	M-062
HI.4765.001.Oat-Plate1	1	GTCAA	FM63	1	F	4	M-063
HI.4765.001.Oat-Plate1	1	GCTCTA	FM64	1	F	5	M-064
HI.4765.001.Oat-Plate1	1	ATCGTA	FM65	1	F	6	M-065
HI.4765.001.Oat-Plate1	1	TAACGA	FM66	1	F	7	M-066
HI.4765.001.Oat-Plate1	1	GGACCTA	FM67	1	F	8	M-067
HI.4765.001.Oat-Plate1	1	CGCTGAT	FM68	1	F	9	M-068
HI.4765.001.Oat-Plate1	1	ACGACTAG	FM69	1	F	10	M-069
HI.4765.001.Oat-Plate1	1	AACCGAGA	FM70	1	F	11	M-070
HI.4765.001.Oat-Plate1	1	GTGAGGGT	FM71	1	F	12	M-071
HI.4765.001.Oat-Plate1	1	CGAT	FM72	1	G	1	M-072
HI.4765.001.Oat-Plate1	1	CTAGG	FM73	1	G	2	M-073
HI.4765.001.Oat-Plate1	1	GGTGT	FM74	1	G	3	M-074
HI.4765.001.Oat-Plate1	1	TAATA	FM75	1	G	4	M-075
HI.4765.001.Oat-Plate1	1	CCACAA	FM76	1	G	5	M-076
HI.4765.001.Oat-Plate1	1	CATCGT	FM77	1	G	6	M-077
HI.4765.001.Oat-Plate1	1	TGGCTA	FM78	1	G	7	M-078
HI.4765.001.Oat-Plate1	1	GTCGATT	FM79	1	G	8	M-079
HI.4765.001.Oat-Plate1	1	CGGTAGA	FM80	1	G	9	M-080
HI.4765.001.Oat-Plate1	1	TAGCATGG	FM81	1	G	10	M-081
HI.4765.001.Oat-Plate1	1	ACAGGGA	FM82	1	G	11	M-082
HI.4765.001.Oat-Plate1	1	TATCGGGA	FM83	1	G	12	M-083
HI.4765.001.Oat-Plate1	1	GTAA	FM84	1	H	1	M-084
HI.4765.001.Oat-Plate1	1	ACAAA	FM85	1	H	2	M-085
HI.4765.001.Oat-Plate1	1	AGGAT	FM86	1	H	3	M-086
HI.4765.001.Oat-Plate1	1	TACAT	FM87	1	H	4	M-087
HI.4765.001.Oat-Plate1	1	CTTCCA	FM88	1	H	5	M-088
HI.4765.001.Oat-Plate1	1	CGCGGT	FM674	1	H	6	M-674
HI.4765.001.Oat-Plate1	1	TATTTTT	FM688	1	H	7	M-688
HI.4765.001.Oat-Plate1	1	AACGCCT	FM701	1	H	8	M-701
HI.4765.002.Oat-Plate2	1	CTCG	FM674	2	A	1	M-674
HI.4765.002.Oat-Plate2	1	AGCG	FM688	2	A	2	M-688
HI.4765.002.Oat-Plate2	1	TTCTG	FM701	2	A	3	M-701
HI.4765.002.Oat-Plate2	1	ATTGA	FM89	2	A	4	M-089
HI.4765.002.Oat-Plate2	1	TCGTT	FM90	2	A	5	M-090
HI.4765.002.Oat-Plate2	1	GAGATA	FM91	2	A	6	M-091

HI.4765.002.Oat-Plate2	1	CTATTA	FM92	2	A	7	M-092
HI.4765.002.Oat-Plate2	1	CTTGCTT	FM93	2	A	8	M-093
HI.4765.002.Oat-Plate2	1	AATATGG	FM94	2	A	9	M-094
HI.4765.002.Oat-Plate2	1	GCGGAAT	FM95	2	A	10	M-095
HI.4765.002.Oat-Plate2	1	TGCAAGGA	FM96	2	A	11	M-096
HI.4765.002.Oat-Plate2	1	CCATGGGT	FM97	2	A	12	M-097
HI.4765.002.Oat-Plate2	1	TGCA	FM98	2	B	1	M-098
HI.4765.002.Oat-Plate2	1	GATG	FM711	2	B	2	Blank
HI.4765.002.Oat-Plate2	1	AGCCG	FM99	2	B	3	M-099
HI.4765.002.Oat-Plate2	1	CATCT	FM100	2	B	4	M-100
HI.4765.002.Oat-Plate2	1	GGTTGT	FM101	2	B	5	M-101
HI.4765.002.Oat-Plate2	1	ATGCCT	FM102	2	B	6	M-102
HI.4765.002.Oat-Plate2	1	GCCAGT	FM103	2	B	7	M-103
HI.4765.002.Oat-Plate2	1	ATGAAAG	FM104	2	B	8	M-104
HI.4765.002.Oat-Plate2	1	ACGTGTT	FM105	2	B	9	M-105
HI.4765.002.Oat-Plate2	1	TAGCGGA	FM106	2	B	10	M-106
HI.4765.002.Oat-Plate2	1	TGGTACGT	FM107	2	B	11	M-107
HI.4765.002.Oat-Plate2	1	CGCGGAGA	FM108	2	B	12	M-108
HI.4765.002.Oat-Plate2	1	ACTA	FM109	2	C	1	M-109
HI.4765.002.Oat-Plate2	1	TCAG	FM110	2	C	2	M-110
HI.4765.002.Oat-Plate2	1	GTATT	FM111	2	C	3	M-111
HI.4765.002.Oat-Plate2	1	CCTAG	FM112	2	C	4	M-112
HI.4765.002.Oat-Plate2	1	CCACGT	FM113	2	C	5	M-113
HI.4765.002.Oat-Plate2	1	AGTGGA	FM114	2	C	6	M-114
HI.4765.002.Oat-Plate2	1	GGAAGA	FM115	2	C	7	M-115
HI.4765.002.Oat-Plate2	1	AAAAGTT	FM116	2	C	8	M-116
HI.4765.002.Oat-Plate2	1	ATTAATT	FM117	2	C	9	M-117
HI.4765.002.Oat-Plate2	1	TCGAAGA	FM118	2	C	10	M-118
HI.4765.002.Oat-Plate2	1	TCTCAGTG	FM119	2	C	11	M-119
HI.4765.002.Oat-Plate2	1	CGTGTGGT	FM120	2	C	12	M-120
HI.4765.002.Oat-Plate2	1	CAGA	FM121	2	D	1	M-121
HI.4765.002.Oat-Plate2	1	TGCGA	FM122	2	D	2	M-122
HI.4765.002.Oat-Plate2	1	CTGTA	FM123	2	D	3	M-123
HI.4765.002.Oat-Plate2	1	GAGGA	FM124	2	D	4	M-124
HI.4765.002.Oat-Plate2	1	TTCAGA	FM125	2	D	5	M-125
HI.4765.002.Oat-Plate2	1	ACCTAA	FM126	2	D	6	M-126
HI.4765.002.Oat-Plate2	1	GTACTT	FM127	2	D	7	M-127
HI.4765.002.Oat-Plate2	1	GAATTCA	FM128	2	D	8	M-128
HI.4765.002.Oat-Plate2	1	ATTGGAT	FM129	2	D	9	M-129
HI.4765.002.Oat-Plate2	1	TCTGTGA	FM130	2	D	10	M-130
HI.4765.002.Oat-Plate2	1	CGCGATAT	FM131	2	D	11	M-131
HI.4765.002.Oat-Plate2	1	GCTGTGGA	FM132	2	D	12	M-132
HI.4765.002.Oat-Plate2	1	AACT	FM133	2	E	1	M-133
HI.4765.002.Oat-Plate2	1	CGCTT	FM134	2	E	2	M-134
HI.4765.002.Oat-Plate2	1	ACCGT	FM135	2	E	3	M-135
HI.4765.002.Oat-Plate2	1	GGAAG	FM136	2	E	4	M-136
HI.4765.002.Oat-Plate2	1	TAGGAA	FM137	2	E	5	M-137
HI.4765.002.Oat-Plate2	1	ATATGT	FM138	2	E	6	M-138
HI.4765.002.Oat-Plate2	1	GTTGAA	FM139	2	E	7	M-139
HI.4765.002.Oat-Plate2	1	GAACTTG	FM140	2	E	8	M-140

HI.4765.002.Oat-Plate2	1	CATAAGT	FM141	2	E	9	M-141
HI.4765.002.Oat-Plate2	1	TGCTGGA	FM142	2	E	10	M-142
HI.4765.002.Oat-Plate2	1	CGCCTTAT	FM143	2	E	11	M-143
HI.4765.002.Oat-Plate2	1	GGATTGGT	FM144	2	E	12	M-144
HI.4765.002.Oat-Plate2	1	GCGT	FM145	2	F	1	M-145
HI.4765.002.Oat-Plate2	1	TCACG	FM146	2	F	2	M-146
HI.4765.002.Oat-Plate2	1	GCTTA	FM147	2	F	3	M-147
HI.4765.002.Oat-Plate2	1	GTCAA	FM148	2	F	4	M-148
HI.4765.002.Oat-Plate2	1	GCTCTA	FM149	2	F	5	M-149
HI.4765.002.Oat-Plate2	1	ATCGTA	FM150	2	F	6	M-150
HI.4765.002.Oat-Plate2	1	TAACGA	FM151	2	F	7	M-151
HI.4765.002.Oat-Plate2	1	GGACCTA	FM152	2	F	8	M-152
HI.4765.002.Oat-Plate2	1	CGCTGAT	FM153	2	F	9	M-153
HI.4765.002.Oat-Plate2	1	ACGACTAG	FM154	2	F	10	M-154
HI.4765.002.Oat-Plate2	1	AACCGAGA	FM155	2	F	11	M-155
HI.4765.002.Oat-Plate2	1	GTGAGGGT	FM156	2	F	12	M-156
HI.4765.002.Oat-Plate2	1	CGAT	FM157	2	G	1	M-157
HI.4765.002.Oat-Plate2	1	CTAGG	FM158	2	G	2	M-158
HI.4765.002.Oat-Plate2	1	GGTGT	FM159	2	G	3	M-159
HI.4765.002.Oat-Plate2	1	TAATA	FM160	2	G	4	M-160
HI.4765.002.Oat-Plate2	1	CCACAA	FM161	2	G	5	M-161
HI.4765.002.Oat-Plate2	1	CATCGT	FM162	2	G	6	M-162
HI.4765.002.Oat-Plate2	1	TGGCTA	FM163	2	G	7	M-163
HI.4765.002.Oat-Plate2	1	GTCGATT	FM164	2	G	8	M-164
HI.4765.002.Oat-Plate2	1	CGGTAGA	FM165	2	G	9	M-165
HI.4765.002.Oat-Plate2	1	TAGCATGG	FM166	2	G	10	M-166
HI.4765.002.Oat-Plate2	1	ACAGGGA	FM167	2	G	11	M-167
HI.4765.002.Oat-Plate2	1	TATCGGGA	FM168	2	G	12	M-168
HI.4765.002.Oat-Plate2	1	GTAA	FM169	2	H	1	M-169
HI.4765.002.Oat-Plate2	1	ACAAA	FM170	2	H	2	M-170
HI.4765.002.Oat-Plate2	1	AGGAT	FM171	2	H	3	M-171
HI.4765.002.Oat-Plate2	1	TACAT	FM172	2	H	4	M-172
HI.4765.002.Oat-Plate2	1	CTTCCA	FM173	2	H	5	M-173
HI.4765.002.Oat-Plate2	1	CGCGGT	FM174	2	H	6	M-174
HI.4765.002.Oat-Plate2	1	TATTTTT	FM175	2	H	7	M-175
HI.4765.002.Oat-Plate2	1	AACGCCT	FM176	2	H	8	M-176
HI.4765.003.Oat-Plate3	1	CTCG	FM177	3	A	1	M-177
HI.4765.003.Oat-Plate3	1	AGCG	FM178	3	A	2	M-178
HI.4765.003.Oat-Plate3	1	TTCTG	FM179	3	A	3	M-179
HI.4765.003.Oat-Plate3	1	ATTGA	FM180	3	A	4	M-180
HI.4765.003.Oat-Plate3	1	TCGTT	FM181	3	A	5	M-181
HI.4765.003.Oat-Plate3	1	GAGATA	FM182	3	A	6	M-182
HI.4765.003.Oat-Plate3	1	CTATTA	FM183	3	A	7	M-183
HI.4765.003.Oat-Plate3	1	CTTGCTT	FM184	3	A	8	M-184
HI.4765.003.Oat-Plate3	1	AATATGG	FM185	3	A	9	M-185
HI.4765.003.Oat-Plate3	1	GCGGAAT	FM186	3	A	10	M-186
HI.4765.003.Oat-Plate3	1	TGCAAGGA	FM187	3	A	11	M-187
HI.4765.003.Oat-Plate3	1	CCATGGGT	FM188	3	A	12	M-188
HI.4765.003.Oat-Plate3	1	TGCA	FM189	3	B	1	M-189
HI.4765.003.Oat-Plate3	1	GATG	FM190	3	B	2	M-190

HI.4765.003.Oat-Plate3	1	AGCCG	FM191	3	B	3	M-191
HI.4765.003.Oat-Plate3	1	CATCT	FM192	3	B	4	M-192
HI.4765.003.Oat-Plate3	1	GGTTGT	FM193	3	B	5	M-193
HI.4765.003.Oat-Plate3	1	ATGCCT	FM194	3	B	6	M-194
HI.4765.003.Oat-Plate3	1	GCCAGT	FM195	3	B	7	M-195
HI.4765.003.Oat-Plate3	1	ATGAAAG	FM196	3	B	8	M-196
HI.4765.003.Oat-Plate3	1	ACGTGTT	FM197	3	B	9	M-197
HI.4765.003.Oat-Plate3	1	TAGCGGA	FM198	3	B	10	M-198
HI.4765.003.Oat-Plate3	1	TGGTACGT	FM199	3	B	11	M-199
HI.4765.003.Oat-Plate3	1	CGCGGAGA	FM200	3	B	12	M-200
HI.4765.003.Oat-Plate3	1	ACTA	FM201	3	C	1	M-201
HI.4765.003.Oat-Plate3	1	TCAG	FM202	3	C	2	M-202
HI.4765.003.Oat-Plate3	1	GTATT	FM712	3	C	3	Blank
HI.4765.003.Oat-Plate3	1	CCTAG	FM674	3	C	4	M-674
HI.4765.003.Oat-Plate3	1	CCACGT	FM688	3	C	5	M-688
HI.4765.003.Oat-Plate3	1	AGTGGA	FM701	3	C	6	M-701
HI.4765.003.Oat-Plate3	1	GGAAGA	FM203	3	C	7	M-203
HI.4765.003.Oat-Plate3	1	AAAAGTT	FM204	3	C	8	M-204
HI.4765.003.Oat-Plate3	1	ATTAATT	FM205	3	C	9	M-205
HI.4765.003.Oat-Plate3	1	TCGAAGA	FM206	3	C	10	M-206
HI.4765.003.Oat-Plate3	1	TCTCAGTG	FM207	3	C	11	M-207
HI.4765.003.Oat-Plate3	1	CGTGTGGT	FM208	3	C	12	M-208
HI.4765.003.Oat-Plate3	1	CAGA	FM209	3	D	1	M-209
HI.4765.003.Oat-Plate3	1	TGCGA	FM210	3	D	2	M-210
HI.4765.003.Oat-Plate3	1	CTGTA	FM211	3	D	3	M-211
HI.4765.003.Oat-Plate3	1	GAGGA	FM212	3	D	4	M-212
HI.4765.003.Oat-Plate3	1	TTCAGA	FM213	3	D	5	M-213
HI.4765.003.Oat-Plate3	1	ACCTAA	FM214	3	D	6	M-214
HI.4765.003.Oat-Plate3	1	GTACTT	FM215	3	D	7	M-215
HI.4765.003.Oat-Plate3	1	GAATTCA	FM216	3	D	8	M-216
HI.4765.003.Oat-Plate3	1	ATTGGAT	FM217	3	D	9	M-217
HI.4765.003.Oat-Plate3	1	TCTGTGA	FM218	3	D	10	M-218
HI.4765.003.Oat-Plate3	1	CGCGATAT	FM219	3	D	11	M-219
HI.4765.003.Oat-Plate3	1	GCTGTGGA	FM220	3	D	12	M-220
HI.4765.003.Oat-Plate3	1	AACT	FM221	3	E	1	M-221
HI.4765.003.Oat-Plate3	1	CGCTT	FM222	3	E	2	M-222
HI.4765.003.Oat-Plate3	1	ACCGT	FM223	3	E	3	M-223
HI.4765.003.Oat-Plate3	1	GGAAG	FM224	3	E	4	M-224
HI.4765.003.Oat-Plate3	1	TAGGAA	FM225	3	E	5	M-225
HI.4765.003.Oat-Plate3	1	ATATGT	FM226	3	E	6	M-226
HI.4765.003.Oat-Plate3	1	GTTGAA	FM227	3	E	7	M-227
HI.4765.003.Oat-Plate3	1	GAACTTG	FM228	3	E	8	M-228
HI.4765.003.Oat-Plate3	1	CATAAGT	FM229	3	E	9	M-229
HI.4765.003.Oat-Plate3	1	TGCTGGA	FM230	3	E	10	M-230
HI.4765.003.Oat-Plate3	1	CGCCTTAT	FM231	3	E	11	M-231
HI.4765.003.Oat-Plate3	1	GGATTGGT	FM232	3	E	12	M-232
HI.4765.003.Oat-Plate3	1	GCGT	FM233	3	F	1	M-233
HI.4765.003.Oat-Plate3	1	TCACG	FM234	3	F	2	M-234
HI.4765.003.Oat-Plate3	1	GCTTA	FM235	3	F	3	M-235
HI.4765.003.Oat-Plate3	1	GTCAA	FM236	3	F	4	M-236

HI.4765.003.Oat-Plate3	1	GCTCTA	FM237	3	F	5	M-237
HI.4765.003.Oat-Plate3	1	ATCGTA	FM238	3	F	6	M-238
HI.4765.003.Oat-Plate3	1	TAACGA	FM239	3	F	7	M-239
HI.4765.003.Oat-Plate3	1	GGACCTA	FM240	3	F	8	M-240
HI.4765.003.Oat-Plate3	1	CGCTGAT	FM241	3	F	9	M-241
HI.4765.003.Oat-Plate3	1	ACGACTAG	FM242	3	F	10	M-242
HI.4765.003.Oat-Plate3	1	AACCGAGA	FM243	3	F	11	M-243
HI.4765.003.Oat-Plate3	1	GTGAGGGT	FM244	3	F	12	M-244
HI.4765.003.Oat-Plate3	1	CGAT	FM245	3	G	1	M-245
HI.4765.003.Oat-Plate3	1	CTAGG	FM246	3	G	2	M-246
HI.4765.003.Oat-Plate3	1	GGTGT	FM247	3	G	3	M-247
HI.4765.003.Oat-Plate3	1	TAATA	FM248	3	G	4	M-248
HI.4765.003.Oat-Plate3	1	CCACAA	FM249	3	G	5	M-249
HI.4765.003.Oat-Plate3	1	CATCGT	FM250	3	G	6	M-250
HI.4765.003.Oat-Plate3	1	TGGCTA	FM251	3	G	7	M-251
HI.4765.003.Oat-Plate3	1	GTCGATT	FM252	3	G	8	M-252
HI.4765.003.Oat-Plate3	1	CGGTAGA	FM253	3	G	9	M-253
HI.4765.003.Oat-Plate3	1	TAGCATGG	FM254	3	G	10	M-254
HI.4765.003.Oat-Plate3	1	ACAGGGA	FM255	3	G	11	M-255
HI.4765.003.Oat-Plate3	1	TATCGGGA	FM256	3	G	12	M-256
HI.4765.003.Oat-Plate3	1	GTAA	FM257	3	H	1	M-257
HI.4765.003.Oat-Plate3	1	ACAAA	FM258	3	H	2	M-258
HI.4765.003.Oat-Plate3	1	AGGAT	FM259	3	H	3	M-259
HI.4765.003.Oat-Plate3	1	TACAT	FM260	3	H	4	M-260
HI.4765.003.Oat-Plate3	1	CTTCCA	FM261	3	H	5	M-261
HI.4765.003.Oat-Plate3	1	CGCGGT	FM262	3	H	6	M-262
HI.4765.003.Oat-Plate3	1	TATTTTT	FM263	3	H	7	M-263
HI.4765.003.Oat-Plate3	1	AACGCCT	FM264	3	H	8	M-264
HI.4765.004.Oat-Plate4	1	CTCG	FM265	4	A	1	M-265
HI.4765.004.Oat-Plate4	1	AGCG	FM266	4	A	2	M-266
HI.4765.004.Oat-Plate4	1	TTCTG	FM267	4	A	3	M-267
HI.4765.004.Oat-Plate4	1	ATTGA	FM268	4	A	4	M-268
HI.4765.004.Oat-Plate4	1	TCGTT	FM269	4	A	5	M-269
HI.4765.004.Oat-Plate4	1	GAGATA	FM270	4	A	6	M-270
HI.4765.004.Oat-Plate4	1	CTATTA	FM271	4	A	7	M-271
HI.4765.004.Oat-Plate4	1	CTTGCTT	FM272	4	A	8	M-272
HI.4765.004.Oat-Plate4	1	AATATGG	FM273	4	A	9	M-273
HI.4765.004.Oat-Plate4	1	GCGGAAT	FM274	4	A	10	M-274
HI.4765.004.Oat-Plate4	1	TGCAAGGA	FM275	4	A	11	M-275
HI.4765.004.Oat-Plate4	1	CCATGGGT	FM276	4	A	12	M-276
HI.4765.004.Oat-Plate4	1	TGCA	FM277	4	B	1	M-277
HI.4765.004.Oat-Plate4	1	GATG	FM278	4	B	2	M-278
HI.4765.004.Oat-Plate4	1	AGCCG	FM279	4	B	3	M-279
HI.4765.004.Oat-Plate4	1	CATCT	FM280	4	B	4	M-280
HI.4765.004.Oat-Plate4	1	GGTTGT	FM281	4	B	5	M-281
HI.4765.004.Oat-Plate4	1	ATGCCT	FM282	4	B	6	M-282
HI.4765.004.Oat-Plate4	1	GCCAGT	FM283	4	B	7	M-283
HI.4765.004.Oat-Plate4	1	ATGAAAG	FM284	4	B	8	M-284
HI.4765.004.Oat-Plate4	1	ACGTGTT	FM285	4	B	9	M-285
HI.4765.004.Oat-Plate4	1	TAGCGGA	FM286	4	B	10	M-286

HI.4765.004.Oat-Plate4	1	TGGTACGT	FM287	4	B	11	M-287
HI.4765.004.Oat-Plate4	1	CGCGGAGA	FM288	4	B	12	M-288
HI.4765.004.Oat-Plate4	1	ACTA	FM289	4	C	1	M-289
HI.4765.004.Oat-Plate4	1	TCAG	FM290	4	C	2	M-290
HI.4765.004.Oat-Plate4	1	GTATT	FM291	4	C	3	M-291
HI.4765.004.Oat-Plate4	1	CCTAG	FM292	4	C	4	M-292
HI.4765.004.Oat-Plate4	1	CCACGT	FM293	4	C	5	M-293
HI.4765.004.Oat-Plate4	1	AGTGGA	FM294	4	C	6	M-294
HI.4765.004.Oat-Plate4	1	GGAAGA	FM295	4	C	7	M-295
HI.4765.004.Oat-Plate4	1	AAAAGTT	FM296	4	C	8	M-296
HI.4765.004.Oat-Plate4	1	ATTAATT	FM297	4	C	9	M-297
HI.4765.004.Oat-Plate4	1	TCGAAGA	FM298	4	C	10	M-298
HI.4765.004.Oat-Plate4	1	TCTCAGTG	FM299	4	C	11	M-299
HI.4765.004.Oat-Plate4	1	CGTGTGGT	FM300	4	C	12	M-300
HI.4765.004.Oat-Plate4	1	CAGA	FM674	4	D	1	M-674
HI.4765.004.Oat-Plate4	1	TGCGA	FM688	4	D	2	M-688
HI.4765.004.Oat-Plate4	1	CTGTA	FM701	4	D	3	M-701
HI.4765.004.Oat-Plate4	1	GAGGA	FM713	4	D	4	Blank
HI.4765.004.Oat-Plate4	1	TTCAGA	FM301	4	D	5	M-301
HI.4765.004.Oat-Plate4	1	ACCTAA	FM302	4	D	6	M-302
HI.4765.004.Oat-Plate4	1	GTACTT	FM303	4	D	7	M-320
HI.4765.004.Oat-Plate4	1	GAATTCA	FM304	4	D	8	M-303
HI.4765.004.Oat-Plate4	1	ATTGGAT	FM305	4	D	9	M-304
HI.4765.004.Oat-Plate4	1	TCTGTGA	FM306	4	D	10	M-305
HI.4765.004.Oat-Plate4	1	CGCGATAT	FM307	4	D	11	M-306
HI.4765.004.Oat-Plate4	1	GCTGTGGA	FM308	4	D	12	M-307
HI.4765.004.Oat-Plate4	1	AACT	FM309	4	E	1	M-308
HI.4765.004.Oat-Plate4	1	CGCTT	FM310	4	E	2	M-309
HI.4765.004.Oat-Plate4	1	ACCGT	FM311	4	E	3	M-310
HI.4765.004.Oat-Plate4	1	GGAAG	FM312	4	E	4	M-311
HI.4765.004.Oat-Plate4	1	TAGGAA	FM313	4	E	5	M-312
HI.4765.004.Oat-Plate4	1	ATATGT	FM314	4	E	6	M-313
HI.4765.004.Oat-Plate4	1	GTTGAA	FM315	4	E	7	M-314
HI.4765.004.Oat-Plate4	1	GAACTTG	FM316	4	E	8	M-315
HI.4765.004.Oat-Plate4	1	CATAAGT	FM317	4	E	9	M-316
HI.4765.004.Oat-Plate4	1	TGCTGGA	FM318	4	E	10	M-317
HI.4765.004.Oat-Plate4	1	CGCCTTAT	FM319	4	E	11	M-318
HI.4765.004.Oat-Plate4	1	GGATTGGT	FM320	4	E	12	M-319
HI.4765.004.Oat-Plate4	1	GCGT	FM321	4	F	1	M-321
HI.4765.004.Oat-Plate4	1	TCACG	FM322	4	F	2	M-322
HI.4765.004.Oat-Plate4	1	GCTTA	FM323	4	F	3	M-323
HI.4765.004.Oat-Plate4	1	GTCAA	FM324	4	F	4	M-324
HI.4765.004.Oat-Plate4	1	GCTCTA	FM325	4	F	5	M-325
HI.4765.004.Oat-Plate4	1	ATCGTA	FM326	4	F	6	M-326
HI.4765.004.Oat-Plate4	1	TAACGA	FM327	4	F	7	M-327
HI.4765.004.Oat-Plate4	1	GGACCTA	FM328	4	F	8	M-328
HI.4765.004.Oat-Plate4	1	CGCTGAT	FM329	4	F	9	M-329
HI.4765.004.Oat-Plate4	1	ACGACTAG	FM330	4	F	10	M-330
HI.4765.004.Oat-Plate4	1	AACCGAGA	FM331	4	F	11	M-331
HI.4765.004.Oat-Plate4	1	GTGAGGGT	FM332	4	F	12	M-332

HI.4765.004.Oat-Plate4	1	CGAT	FM333	4	G	1	M-333
HI.4765.004.Oat-Plate4	1	CTAGG	FM334	4	G	2	M-334
HI.4765.004.Oat-Plate4	1	GGTGT	FM335	4	G	3	M-335
HI.4765.004.Oat-Plate4	1	TAATA	FM336	4	G	4	M-336
HI.4765.004.Oat-Plate4	1	CCACAA	FM337	4	G	5	M-337
HI.4765.004.Oat-Plate4	1	CATCGT	FM338	4	G	6	M-338
HI.4765.004.Oat-Plate4	1	TGGCTA	FM339	4	G	7	M-339
HI.4765.004.Oat-Plate4	1	GTCGATT	FM340	4	G	8	M-340
HI.4765.004.Oat-Plate4	1	CGGTAGA	FM341	4	G	9	M-341
HI.4765.004.Oat-Plate4	1	TAGCATGG	FM342	4	G	10	M-342
HI.4765.004.Oat-Plate4	1	ACAGGGA	FM343	4	G	11	M-343
HI.4765.004.Oat-Plate4	1	TATCGGGA	FM344	4	G	12	M-344
HI.4765.004.Oat-Plate4	1	GTAA	FM345	4	H	1	M-345
HI.4765.004.Oat-Plate4	1	ACAAA	FM346	4	H	2	M-346
HI.4765.004.Oat-Plate4	1	AGGAT	FM347	4	H	3	M-347
HI.4765.004.Oat-Plate4	1	TACAT	FM348	4	H	4	M-348
HI.4765.004.Oat-Plate4	1	CTTCCA	FM349	4	H	5	M-349
HI.4765.004.Oat-Plate4	1	CGCGGT	FM350	4	H	6	M-350
HI.4765.004.Oat-Plate4	1	TATTTTT	FM351	4	H	7	M-351
HI.4765.004.Oat-Plate4	1	AACGCCT	FM352	4	H	8	M-352
HI.4765.005.Oat-Plate5	1	CTCG	FM353	5	A	1	M-353
HI.4765.005.Oat-Plate5	1	AGCG	FM354	5	A	2	M-354
HI.4765.005.Oat-Plate5	1	TTCTG	FM355	5	A	3	M-355
HI.4765.005.Oat-Plate5	1	ATTGA	FM356	5	A	4	M-356
HI.4765.005.Oat-Plate5	1	TCGTT	FM357	5	A	5	M-357
HI.4765.005.Oat-Plate5	1	GAGATA	FM358	5	A	6	M-358
HI.4765.005.Oat-Plate5	1	CTATTA	FM359	5	A	7	M-359
HI.4765.005.Oat-Plate5	1	CTTGCTT	FM360	5	A	8	M-360
HI.4765.005.Oat-Plate5	1	AATATGG	FM361	5	A	9	M-361
HI.4765.005.Oat-Plate5	1	GCGGAAT	FM362	5	A	10	M-362
HI.4765.005.Oat-Plate5	1	TGCAAGGA	FM363	5	A	11	M-363
HI.4765.005.Oat-Plate5	1	CCATGGGT	FM364	5	A	12	M-364
HI.4765.005.Oat-Plate5	1	TGCA	FM365	5	B	1	M-365
HI.4765.005.Oat-Plate5	1	GATG	FM366	5	B	2	M-366
HI.4765.005.Oat-Plate5	1	AGCCG	FM367	5	B	3	M-367
HI.4765.005.Oat-Plate5	1	CATCT	FM368	5	B	4	M-368
HI.4765.005.Oat-Plate5	1	GGTTGT	FM369	5	B	5	M-369
HI.4765.005.Oat-Plate5	1	ATGCCT	FM370	5	B	6	M-370
HI.4765.005.Oat-Plate5	1	GCCAGT	FM371	5	B	7	M-371
HI.4765.005.Oat-Plate5	1	ATGAAAG	FM372	5	B	8	M-372
HI.4765.005.Oat-Plate5	1	ACGTGTT	FM373	5	B	9	M-373
HI.4765.005.Oat-Plate5	1	TAGCGGA	FM374	5	B	10	M-374
HI.4765.005.Oat-Plate5	1	TGGTACGT	FM375	5	B	11	M-375
HI.4765.005.Oat-Plate5	1	CGCGGAGA	FM376	5	B	12	M-376
HI.4765.005.Oat-Plate5	1	ACTA	FM377	5	C	1	M-377
HI.4765.005.Oat-Plate5	1	TCAG	FM378	5	C	2	M-378
HI.4765.005.Oat-Plate5	1	GTATT	FM379	5	C	3	M-379
HI.4765.005.Oat-Plate5	1	CCTAG	FM380	5	C	4	M-380
HI.4765.005.Oat-Plate5	1	CCACGT	FM381	5	C	5	M-381
HI.4765.005.Oat-Plate5	1	AGTGGA	FM382	5	C	6	M-382

HI.4765.005.Oat-Plate5	1	GGAAGA	FM383	5	C	7	M-383
HI.4765.005.Oat-Plate5	1	AAAAGTT	FM384	5	C	8	M-384
HI.4765.005.Oat-Plate5	1	ATTAATT	FM385	5	C	9	M-385
HI.4765.005.Oat-Plate5	1	TCGAAGA	FM386	5	C	10	M-386
HI.4765.005.Oat-Plate5	1	TCTCAGTG	FM387	5	C	11	M-387
HI.4765.005.Oat-Plate5	1	CGTGTGGT	FM388	5	C	12	M-388
HI.4765.005.Oat-Plate5	1	CAGA	FM389	5	D	1	M-389
HI.4765.005.Oat-Plate5	1	TGCGA	FM390	5	D	2	M-390
HI.4765.005.Oat-Plate5	1	CTGTA	FM391	5	D	3	M-391
HI.4765.005.Oat-Plate5	1	GAGGA	FM392	5	D	4	M-392
HI.4765.005.Oat-Plate5	1	TTCAGA	FM393	5	D	5	M-393
HI.4765.005.Oat-Plate5	1	ACCTAA	FM394	5	D	6	M-394
HI.4765.005.Oat-Plate5	1	GTACTT	FM395	5	D	7	M-395
HI.4765.005.Oat-Plate5	1	GAATTCA	FM396	5	D	8	M-396
HI.4765.005.Oat-Plate5	1	ATTGGAT	FM397	5	D	9	M-397
HI.4765.005.Oat-Plate5	1	TCTGTGA	FM398	5	D	10	M-398
HI.4765.005.Oat-Plate5	1	CGCGATAT	FM399	5	D	11	M-399
HI.4765.005.Oat-Plate5	1	GCTGTGGA	FM400	5	D	12	M-400
HI.4765.005.Oat-Plate5	1	AACT	FM401	5	E	1	M-401
HI.4765.005.Oat-Plate5	1	CGCTT	FM402	5	E	2	M-402
HI.4765.005.Oat-Plate5	1	ACCGT	FM403	5	E	3	M-403
HI.4765.005.Oat-Plate5	1	GGAAG	FM404	5	E	4	M-404
HI.4765.005.Oat-Plate5	1	TAGGAA	FM714	5	E	5	Blank
HI.4765.005.Oat-Plate5	1	ATATGT	FM674	5	E	6	M-674
HI.4765.005.Oat-Plate5	1	GTTGAA	FM688	5	E	7	M-688
HI.4765.005.Oat-Plate5	1	GAACTTG	FM701	5	E	8	M-701
HI.4765.005.Oat-Plate5	1	CATAAGT	FM405	5	E	9	M-405
HI.4765.005.Oat-Plate5	1	TGCTGGA	FM406	5	E	10	M-406
HI.4765.005.Oat-Plate5	1	CGCCTTAT	FM407	5	E	11	M-407
HI.4765.005.Oat-Plate5	1	GGATTGGT	FM408	5	E	12	M-408
HI.4765.005.Oat-Plate5	1	GCGT	FM409	5	F	1	M-409
HI.4765.005.Oat-Plate5	1	TCACG	FM410	5	F	2	M-410
HI.4765.005.Oat-Plate5	1	GCTTA	FM411	5	F	3	M-411
HI.4765.005.Oat-Plate5	1	GTCAA	FM412	5	F	4	M-412
HI.4765.005.Oat-Plate5	1	GCTCTA	FM413	5	F	5	M-413
HI.4765.005.Oat-Plate5	1	ATCGTA	FM414	5	F	6	M-414
HI.4765.005.Oat-Plate5	1	TAACGA	FM415	5	F	7	M-415
HI.4765.005.Oat-Plate5	1	GGACCTA	FM416	5	F	8	M-416
HI.4765.005.Oat-Plate5	1	CGCTGAT	FM417	5	F	9	M-417
HI.4765.005.Oat-Plate5	1	ACGACTAG	FM418	5	F	10	M-418
HI.4765.005.Oat-Plate5	1	AACCGAGA	FM419	5	F	11	M-419
HI.4765.005.Oat-Plate5	1	GTGAGGGT	FM420	5	F	12	M-420
HI.4765.005.Oat-Plate5	1	CGAT	FM421	5	G	1	M-421
HI.4765.005.Oat-Plate5	1	CTAGG	FM422	5	G	2	M-422
HI.4765.005.Oat-Plate5	1	GGTGT	FM423	5	G	3	M-423
HI.4765.005.Oat-Plate5	1	TAATA	FM424	5	G	4	M-424
HI.4765.005.Oat-Plate5	1	CCACAA	FM425	5	G	5	M-425
HI.4765.005.Oat-Plate5	1	CATCGT	FM426	5	G	6	M-426
HI.4765.005.Oat-Plate5	1	TGGCTA	FM427	5	G	7	M-427
HI.4765.005.Oat-Plate5	1	GTCGATT	FM428	5	G	8	M-428

HI.4765.005.Oat-Plate5	1	CGGTAGA	FM429	5	G	9	M-429
HI.4765.005.Oat-Plate5	1	TAGCATGG	FM430	5	G	10	M-430
HI.4765.005.Oat-Plate5	1	ACAGGGA	FM431	5	G	11	M-431
HI.4765.005.Oat-Plate5	1	TATCGGGA	FM432	5	G	12	M-432
HI.4765.005.Oat-Plate5	1	GTAA	FM433	5	H	1	M-433
HI.4765.005.Oat-Plate5	1	ACAAA	FM434	5	H	2	M-434
HI.4765.005.Oat-Plate5	1	AGGAT	FM435	5	H	3	M-435
HI.4765.005.Oat-Plate5	1	TACAT	FM436	5	H	4	M-436
HI.4765.005.Oat-Plate5	1	CTTCCA	FM437	5	H	5	M-437
HI.4765.005.Oat-Plate5	1	CGCGGT	FM438	5	H	6	M-438
HI.4765.005.Oat-Plate5	1	TATTTTT	FM439	5	H	7	M-439
HI.4765.005.Oat-Plate5	1	AACGCCT	FM440	5	H	8	M-440
HI.4765.006.Oat-Plate6	1	CTCG	FM441	6	A	1	M-441
HI.4765.006.Oat-Plate6	1	AGCG	FM442	6	A	2	M-442
HI.4765.006.Oat-Plate6	1	TTCTG	FM443	6	A	3	M-443
HI.4765.006.Oat-Plate6	1	ATTGA	FM444	6	A	4	M-444
HI.4765.006.Oat-Plate6	1	TCGTT	FM445	6	A	5	M-445
HI.4765.006.Oat-Plate6	1	GAGATA	FM446	6	A	6	M-446
HI.4765.006.Oat-Plate6	1	CTATTA	FM447	6	A	7	M-447
HI.4765.006.Oat-Plate6	1	CTTGCTT	FM448	6	A	8	M-448
HI.4765.006.Oat-Plate6	1	AATATGG	FM449	6	A	9	M-449
HI.4765.006.Oat-Plate6	1	GCGGAAT	FM450	6	A	10	M-450
HI.4765.006.Oat-Plate6	1	TGCAAGGA	FM451	6	A	11	M-451
HI.4765.006.Oat-Plate6	1	CCATGGGT	FM452	6	A	12	M-452
HI.4765.006.Oat-Plate6	1	TGCA	FM453	6	B	1	M-453
HI.4765.006.Oat-Plate6	1	GATG	FM454	6	B	2	M-454
HI.4765.006.Oat-Plate6	1	AGCCG	FM455	6	B	3	M-455
HI.4765.006.Oat-Plate6	1	CATCT	FM456	6	B	4	M-456
HI.4765.006.Oat-Plate6	1	GGTTGT	FM457	6	B	5	M-457
HI.4765.006.Oat-Plate6	1	ATGCCT	FM458	6	B	6	M-458
HI.4765.006.Oat-Plate6	1	GCCAGT	FM459	6	B	7	M-459
HI.4765.006.Oat-Plate6	1	ATGAAAG	FM460	6	B	8	M-460
HI.4765.006.Oat-Plate6	1	ACGTGTT	FM461	6	B	9	M-461
HI.4765.006.Oat-Plate6	1	TAGCGGA	FM462	6	B	10	M-462
HI.4765.006.Oat-Plate6	1	TGGTACGT	FM463	6	B	11	M-463
HI.4765.006.Oat-Plate6	1	CGCGGAGA	FM464	6	B	12	M-464
HI.4765.006.Oat-Plate6	1	ACTA	FM465	6	C	1	M-465
HI.4765.006.Oat-Plate6	1	TCAG	FM466	6	C	2	M-466
HI.4765.006.Oat-Plate6	1	GTATT	FM467	6	C	3	M-467
HI.4765.006.Oat-Plate6	1	CCTAG	FM468	6	C	4	M-468
HI.4765.006.Oat-Plate6	1	CCACGT	FM469	6	C	5	M-469
HI.4765.006.Oat-Plate6	1	AGTGGA	FM470	6	C	6	M-470
HI.4765.006.Oat-Plate6	1	GGAAGA	FM471	6	C	7	M-471
HI.4765.006.Oat-Plate6	1	AAAAGTT	FM472	6	C	8	M-472
HI.4765.006.Oat-Plate6	1	ATTAATT	FM473	6	C	9	M-473
HI.4765.006.Oat-Plate6	1	TCGAAGA	FM474	6	C	10	M-474
HI.4765.006.Oat-Plate6	1	TCTCAGTG	FM475	6	C	11	M-475
HI.4765.006.Oat-Plate6	1	CGTGTGGT	FM476	6	C	12	M-476
HI.4765.006.Oat-Plate6	1	CAGA	FM477	6	D	1	M-477
HI.4765.006.Oat-Plate6	1	TGCGA	FM478	6	D	2	M-478

HI.4765.006.Oat-Plate6	1	CTGTA	FM479	6	D	3	M-479
HI.4765.006.Oat-Plate6	1	GAGGA	FM480	6	D	4	M-480
HI.4765.006.Oat-Plate6	1	TTCAGA	FM481	6	D	5	M-481
HI.4765.006.Oat-Plate6	1	ACCTAA	FM482	6	D	6	M-482
HI.4765.006.Oat-Plate6	1	GTACTT	FM483	6	D	7	M-483
HI.4765.006.Oat-Plate6	1	GAATTCA	FM484	6	D	8	M-484
HI.4765.006.Oat-Plate6	1	ATTGGAT	FM485	6	D	9	M-485
HI.4765.006.Oat-Plate6	1	TCTGTGA	FM486	6	D	10	M-486
HI.4765.006.Oat-Plate6	1	CGCGATAT	FM487	6	D	11	M-487
HI.4765.006.Oat-Plate6	1	GCTGTGGA	FM488	6	D	12	M-488
HI.4765.006.Oat-Plate6	1	AACT	FM489	6	E	1	M-489
HI.4765.006.Oat-Plate6	1	CGCTT	FM490	6	E	2	M-490
HI.4765.006.Oat-Plate6	1	ACCGT	FM491	6	E	3	M-491
HI.4765.006.Oat-Plate6	1	GGAAG	FM492	6	E	4	M-492
HI.4765.006.Oat-Plate6	1	TAGGAA	FM493	6	E	5	M-493
HI.4765.006.Oat-Plate6	1	ATATGT	FM494	6	E	6	M-494
HI.4765.006.Oat-Plate6	1	GTTGAA	FM495	6	E	7	M-495
HI.4765.006.Oat-Plate6	1	GAACTTG	FM496	6	E	8	M-496
HI.4765.006.Oat-Plate6	1	CATAAGT	FM497	6	E	9	M-497
HI.4765.006.Oat-Plate6	1	TGCTGGA	FM498	6	E	10	M-498
HI.4765.006.Oat-Plate6	1	CGCCTTAT	FM499	6	E	11	M-499
HI.4765.006.Oat-Plate6	1	GGATTGGT	FM500	6	E	12	M-500
HI.4765.006.Oat-Plate6	1	GCGT	FM501	6	F	1	M-501
HI.4765.006.Oat-Plate6	1	TCACG	FM502	6	F	2	M-502
HI.4765.006.Oat-Plate6	1	GCTTA	FM503	6	F	3	M-503
HI.4765.006.Oat-Plate6	1	GTCAA	FM504	6	F	4	M-504
HI.4765.006.Oat-Plate6	1	GCTCTA	FM505	6	F	5	M-505
HI.4765.006.Oat-Plate6	1	ATCGTA	FM715	6	F	6	Blank
HI.4765.006.Oat-Plate6	1	TAACGA	FM506	6	F	7	M-506
HI.4765.006.Oat-Plate6	1	GGACCTA	FM507	6	F	8	M-507
HI.4765.006.Oat-Plate6	1	CGCTGAT	FM508	6	F	9	M-508
HI.4765.006.Oat-Plate6	1	ACGACTAG	FM509	6	F	10	M-509
HI.4765.006.Oat-Plate6	1	AACCGAGA	FM510	6	F	11	M-510
HI.4765.006.Oat-Plate6	1	GTGAGGGT	FM511	6	F	12	M-511
HI.4765.006.Oat-Plate6	1	CGAT	FM512	6	G	1	M-512
HI.4765.006.Oat-Plate6	1	CTAGG	FM513	6	G	2	M-513
HI.4765.006.Oat-Plate6	1	GGTGT	FM514	6	G	3	M-514
HI.4765.006.Oat-Plate6	1	TAATA	FM515	6	G	4	M-515
HI.4765.006.Oat-Plate6	1	CCACAA	FM516	6	G	5	M-516
HI.4765.006.Oat-Plate6	1	CATCGT	FM517	6	G	6	M-517
HI.4765.006.Oat-Plate6	1	TGGCTA	FM518	6	G	7	M-518
HI.4765.006.Oat-Plate6	1	GTCGATT	FM519	6	G	8	M-519
HI.4765.006.Oat-Plate6	1	CGGTAGA	FM520	6	G	9	M-520
HI.4765.006.Oat-Plate6	1	TAGCATGG	FM521	6	G	10	M-521
HI.4765.006.Oat-Plate6	1	ACAGGGA	FM522	6	G	11	M-522
HI.4765.006.Oat-Plate6	1	TATCGGGA	FM523	6	G	12	M-523
HI.4765.006.Oat-Plate6	1	GTAA	FM524	6	H	1	M-524
HI.4765.006.Oat-Plate6	1	ACAAA	FM525	6	H	2	M-525
HI.4765.006.Oat-Plate6	1	AGGAT	FM526	6	H	3	M-526
HI.4765.006.Oat-Plate6	1	TACAT	FM527	6	H	4	M-527

HI.4765.006.Oat-Plate6	1	CTTCCA	FM528	6	H	5	M-528
HI.4765.006.Oat-Plate6	1	CGCGGT	FM674	6	H	6	M-674
HI.4765.006.Oat-Plate6	1	TATTTTT	FM688	6	H	7	M-688
HI.4765.006.Oat-Plate6	1	AACGCCT	FM701	6	H	8	M-701
HI.4765.007.Oat-Plate7	1	CTCG	FM709	7	A	1	M-709
HI.4765.007.Oat-Plate7	1	AGCG	FM674	7	A	2	M-674
HI.4765.007.Oat-Plate7	1	TTCTG	FM701	7	A	3	M-701
HI.4765.007.Oat-Plate7	1	ATTGA	FM529	7	A	4	M-529
HI.4765.007.Oat-Plate7	1	TCGTT	FM530	7	A	5	M-530
HI.4765.007.Oat-Plate7	1	GAGATA	FM531	7	A	6	M-531
HI.4765.007.Oat-Plate7	1	CTATTA	FM532	7	A	7	M-532
HI.4765.007.Oat-Plate7	1	CTTGCTT	FM533	7	A	8	M-533
HI.4765.007.Oat-Plate7	1	AATATGG	FM534	7	A	9	M-534
HI.4765.007.Oat-Plate7	1	GCGGAAT	FM535	7	A	10	M-535
HI.4765.007.Oat-Plate7	1	TGCAAGGA	FM536	7	A	11	M-536
HI.4765.007.Oat-Plate7	1	CCATGGGT	FM537	7	A	12	M-537
HI.4765.007.Oat-Plate7	1	TGCA	FM538	7	B	1	M-538
HI.4765.007.Oat-Plate7	1	GATG	FM539	7	B	2	M-539
HI.4765.007.Oat-Plate7	1	AGCCG	FM540	7	B	3	M-540
HI.4765.007.Oat-Plate7	1	CATCT	FM541	7	B	4	M-541
HI.4765.007.Oat-Plate7	1	GGTTGT	FM542	7	B	5	M-542
HI.4765.007.Oat-Plate7	1	ATGCCT	FM543	7	B	6	M-543
HI.4765.007.Oat-Plate7	1	GCCAGT	FM544	7	B	7	M-544
HI.4765.007.Oat-Plate7	1	ATGAAAG	FM545	7	B	8	M-545
HI.4765.007.Oat-Plate7	1	ACGTGTT	FM546	7	B	9	M-546
HI.4765.007.Oat-Plate7	1	TAGCGGA	FM547	7	B	10	M-547
HI.4765.007.Oat-Plate7	1	TGGTACGT	FM548	7	B	11	M-548
HI.4765.007.Oat-Plate7	1	CGCGGAGA	FM549	7	B	12	M-549
HI.4765.007.Oat-Plate7	1	ACTA	FM550	7	C	1	M-550
HI.4765.007.Oat-Plate7	1	TCAG	FM551	7	C	2	M-551
HI.4765.007.Oat-Plate7	1	GTATT	FM552	7	C	3	M-552
HI.4765.007.Oat-Plate7	1	CCTAG	FM553	7	C	4	M-553
HI.4765.007.Oat-Plate7	1	CCACGT	FM554	7	C	5	M-554
HI.4765.007.Oat-Plate7	1	AGTGGA	FM555	7	C	6	M-555
HI.4765.007.Oat-Plate7	1	GGAAGA	FM556	7	C	7	M-556
HI.4765.007.Oat-Plate7	1	AAAAGTT	FM557	7	C	8	M-557
HI.4765.007.Oat-Plate7	1	ATTAATT	FM558	7	C	9	M-558
HI.4765.007.Oat-Plate7	1	TCGAAGA	FM559	7	C	10	M-559
HI.4765.007.Oat-Plate7	1	TCTCAGTG	FM560	7	C	11	M-560
HI.4765.007.Oat-Plate7	1	CGTGTGGT	FM561	7	C	12	M-561
HI.4765.007.Oat-Plate7	1	CAGA	FM562	7	D	1	M-562
HI.4765.007.Oat-Plate7	1	TGCGA	FM563	7	D	2	M-563
HI.4765.007.Oat-Plate7	1	CTGTA	FM564	7	D	3	M-564
HI.4765.007.Oat-Plate7	1	GAGGA	FM565	7	D	4	M-565
HI.4765.007.Oat-Plate7	1	TTCAGA	FM566	7	D	5	M-566
HI.4765.007.Oat-Plate7	1	ACCTAA	FM567	7	D	6	M-567
HI.4765.007.Oat-Plate7	1	GTACTT	FM568	7	D	7	M-568
HI.4765.007.Oat-Plate7	1	GAATTCA	FM569	7	D	8	M-569
HI.4765.007.Oat-Plate7	1	ATTGGAT	FM570	7	D	9	M-570
HI.4765.007.Oat-Plate7	1	TCTGTGA	FM571	7	D	10	M-571

HI.4765.007.Oat-Plate7	1	CGCGATAT	FM572	7	D	11	M-572
HI.4765.007.Oat-Plate7	1	GCTGTGGA	FM573	7	D	12	M-573
HI.4765.007.Oat-Plate7	1	AACT	FM574	7	E	1	M-574
HI.4765.007.Oat-Plate7	1	CGCTT	FM575	7	E	2	M-575
HI.4765.007.Oat-Plate7	1	ACCGT	FM576	7	E	3	M-576
HI.4765.007.Oat-Plate7	1	GGAAG	FM577	7	E	4	M-577
HI.4765.007.Oat-Plate7	1	TAGGAA	FM578	7	E	5	M-578
HI.4765.007.Oat-Plate7	1	ATATGT	FM579	7	E	6	M-579
HI.4765.007.Oat-Plate7	1	GTTGAA	FM580	7	E	7	M-580
HI.4765.007.Oat-Plate7	1	GAACTTG	FM581	7	E	8	M-581
HI.4765.007.Oat-Plate7	1	CATAAGT	FM582	7	E	9	M-582
HI.4765.007.Oat-Plate7	1	TGCTGGA	FM583	7	E	10	M-583
HI.4765.007.Oat-Plate7	1	CGCCTTAT	FM584	7	E	11	M-584
HI.4765.007.Oat-Plate7	1	GGATTGGT	FM585	7	E	12	M-585
HI.4765.007.Oat-Plate7	1	GCGT	FM586	7	F	1	M-586
HI.4765.007.Oat-Plate7	1	TCACG	FM587	7	F	2	M-587
HI.4765.007.Oat-Plate7	1	GCTTA	FM588	7	F	3	M-588
HI.4765.007.Oat-Plate7	1	GTCAA	FM589	7	F	4	M-589
HI.4765.007.Oat-Plate7	1	GCTCTA	FM590	7	F	5	M-590
HI.4765.007.Oat-Plate7	1	ATCGTA	FM591	7	F	6	M-591
HI.4765.007.Oat-Plate7	1	TAACGA	FM592	7	F	7	M-592
HI.4765.007.Oat-Plate7	1	GGACCTA	FM593	7	F	8	M-593
HI.4765.007.Oat-Plate7	1	CGCTGAT	FM594	7	F	9	M-594
HI.4765.007.Oat-Plate7	1	ACGACTAG	FM595	7	F	10	M-595
HI.4765.007.Oat-Plate7	1	AACCGAGA	FM596	7	F	11	M-596
HI.4765.007.Oat-Plate7	1	GTGAGGGT	FM597	7	F	12	M-597
HI.4765.007.Oat-Plate7	1	CGAT	FM598	7	G	1	M-598
HI.4765.007.Oat-Plate7	1	CTAGG	FM599	7	G	2	M-599
HI.4765.007.Oat-Plate7	1	GGTGT	FM600	7	G	3	M-600
HI.4765.007.Oat-Plate7	1	TAATA	FM601	7	G	4	M-601
HI.4765.007.Oat-Plate7	1	CCACAA	FM602	7	G	5	M-602
HI.4765.007.Oat-Plate7	1	CATCGT	FM603	7	G	6	M-603
HI.4765.007.Oat-Plate7	1	TGGCTA	FM604	7	G	7	M-604
HI.4765.007.Oat-Plate7	1	GTCGATT	FM716	7	G	8	Blank
HI.4765.007.Oat-Plate7	1	CGGTAGA	FM605	7	G	9	M-605
HI.4765.007.Oat-Plate7	1	TAGCATGG	FM606	7	G	10	M-606
HI.4765.007.Oat-Plate7	1	ACAGGGA	FM607	7	G	11	M-607
HI.4765.007.Oat-Plate7	1	TATCGGGA	FM608	7	G	12	M-608
HI.4765.007.Oat-Plate7	1	GTAA	FM609	7	H	1	M-609
HI.4765.007.Oat-Plate7	1	ACAAA	FM610	7	H	2	M-610
HI.4765.007.Oat-Plate7	1	AGGAT	FM611	7	H	3	M-611
HI.4765.007.Oat-Plate7	1	TACAT	FM612	7	H	4	M-612
HI.4765.007.Oat-Plate7	1	CTTCCA	FM613	7	H	5	M-613
HI.4765.007.Oat-Plate7	1	CGCGGT	FM614	7	H	6	M-614
HI.4765.007.Oat-Plate7	1	TATTTTT	FM615	7	H	7	M-615
HI.4765.007.Oat-Plate7	1	AACGCCT	FM616	7	H	8	M-616
HI.4765.008.Oat-Plate8	1	CTCG	FM617	8	A	1	M-617
HI.4765.008.Oat-Plate8	1	AGCG	FM618	8	A	2	M-618
HI.4765.008.Oat-Plate8	1	TTCTG	FM619	8	A	3	M-619
HI.4765.008.Oat-Plate8	1	ATTGA	FM620	8	A	4	M-620

HI.4765.008.Oat-Plate8	1	TCGTT	FM621	8	A	5	M-621
HI.4765.008.Oat-Plate8	1	GAGATA	FM622	8	A	6	M-622
HI.4765.008.Oat-Plate8	1	CTATTA	FM623	8	A	7	M-623
HI.4765.008.Oat-Plate8	1	CTTGCTT	FM624	8	A	8	M-624
HI.4765.008.Oat-Plate8	1	AATATGG	FM625	8	A	9	M-625
HI.4765.008.Oat-Plate8	1	GCGGAAT	FM626	8	A	10	M-626
HI.4765.008.Oat-Plate8	1	TGCAAGGA	FM627	8	A	11	M-627
HI.4765.008.Oat-Plate8	1	CCATGGGT	FM628	8	A	12	M-628
HI.4765.008.Oat-Plate8	1	TGCA	FM629	8	B	1	M-629
HI.4765.008.Oat-Plate8	1	GATG	FM630	8	B	2	M-630
HI.4765.008.Oat-Plate8	1	AGCCG	FM631	8	B	3	M-631
HI.4765.008.Oat-Plate8	1	CATCT	FM632	8	B	4	M-632
HI.4765.008.Oat-Plate8	1	GGTTGT	FM633	8	B	5	M-633
HI.4765.008.Oat-Plate8	1	ATGCCT	FM634	8	B	6	M-634
HI.4765.008.Oat-Plate8	1	GCCAGT	FM635	8	B	7	M-635
HI.4765.008.Oat-Plate8	1	ATGAAAG	FM636	8	B	8	M-636
HI.4765.008.Oat-Plate8	1	ACGTGTT	FM637	8	B	9	M-637
HI.4765.008.Oat-Plate8	1	TAGCGGA	FM638	8	B	10	M-638
HI.4765.008.Oat-Plate8	1	TGGTACGT	FM639	8	B	11	M-639
HI.4765.008.Oat-Plate8	1	CGCGGAGA	FM640	8	B	12	M-640
HI.4765.008.Oat-Plate8	1	ACTA	FM641	8	C	1	M-641
HI.4765.008.Oat-Plate8	1	TCAG	FM642	8	C	2	M-642
HI.4765.008.Oat-Plate8	1	GTATT	FM643	8	C	3	M-643
HI.4765.008.Oat-Plate8	1	CCTAG	FM644	8	C	4	M-644
HI.4765.008.Oat-Plate8	1	CCACGT	FM645	8	C	5	M-645
HI.4765.008.Oat-Plate8	1	AGTGGA	FM646	8	C	6	M-646
HI.4765.008.Oat-Plate8	1	GGAAGA	FM647	8	C	7	M-647
HI.4765.008.Oat-Plate8	1	AAAAGTT	FM648	8	C	8	M-648
HI.4765.008.Oat-Plate8	1	ATTAATT	FM649	8	C	9	M-649
HI.4765.008.Oat-Plate8	1	TCGAAGA	FM650	8	C	10	M-650
HI.4765.008.Oat-Plate8	1	TCTCAGTG	FM651	8	C	11	M-651
HI.4765.008.Oat-Plate8	1	CGTGTGGT	FM652	8	C	12	M-652
HI.4765.008.Oat-Plate8	1	CAGA	FM653	8	D	1	M-653
HI.4765.008.Oat-Plate8	1	TGCGA	FM654	8	D	2	M-654
HI.4765.008.Oat-Plate8	1	CTGTA	FM655	8	D	3	M-655
HI.4765.008.Oat-Plate8	1	GAGGA	FM656	8	D	4	M-656
HI.4765.008.Oat-Plate8	1	TTCAGA	FM657	8	D	5	M-657
HI.4765.008.Oat-Plate8	1	ACCTAA	FM658	8	D	6	M-658
HI.4765.008.Oat-Plate8	1	GTACTT	FM659	8	D	7	M-659
HI.4765.008.Oat-Plate8	1	GAATTCA	FM660	8	D	8	M-660
HI.4765.008.Oat-Plate8	1	ATTGGAT	FM661	8	D	9	M-661
HI.4765.008.Oat-Plate8	1	TCTGTGA	FM662	8	D	10	M-662
HI.4765.008.Oat-Plate8	1	CGCGATAT	FM663	8	D	11	M-663
HI.4765.008.Oat-Plate8	1	GCTGTGGA	FM664	8	D	12	M-664
HI.4765.008.Oat-Plate8	1	AACT	FM665	8	E	1	M-665
HI.4765.008.Oat-Plate8	1	CGCTT	FM666	8	E	2	M-666
HI.4765.008.Oat-Plate8	1	ACCGT	FM667	8	E	3	M-667
HI.4765.008.Oat-Plate8	1	GGAAG	FM668	8	E	4	M-668
HI.4765.008.Oat-Plate8	1	TAGGAA	FM669	8	E	5	M-669
HI.4765.008.Oat-Plate8	1	ATATGT	FM670	8	E	6	M-670

HI.4765.008.Oat-Plate8	1	GTTGAA	FM671	8	E	7	M-671
HI.4765.008.Oat-Plate8	1	GAACTTG	FM672	8	E	8	M-672
HI.4765.008.Oat-Plate8	1	CATAAGT	FM673	8	E	9	M-673
HI.4765.008.Oat-Plate8	1	TGCTGGA	FM674	8	E	10	M-674
HI.4765.008.Oat-Plate8	1	CGCCTTAT	FM675	8	E	11	M-675
HI.4765.008.Oat-Plate8	1	GGATTGGT	FM676	8	E	12	M-676
HI.4765.008.Oat-Plate8	1	GCGT	FM677	8	F	1	M-677
HI.4765.008.Oat-Plate8	1	TCACG	FM678	8	F	2	M-678
HI.4765.008.Oat-Plate8	1	GCTTA	FM679	8	F	3	M-679
HI.4765.008.Oat-Plate8	1	GTCAA	FM680	8	F	4	M-680
HI.4765.008.Oat-Plate8	1	GCTCTA	FM681	8	F	5	M-681
HI.4765.008.Oat-Plate8	1	ATCGTA	FM682	8	F	6	M-682
HI.4765.008.Oat-Plate8	1	TAACGA	FM683	8	F	7	M-683
HI.4765.008.Oat-Plate8	1	GGACCTA	FM684	8	F	8	M-684
HI.4765.008.Oat-Plate8	1	CGCTGAT	FM685	8	F	9	M-685
HI.4765.008.Oat-Plate8	1	ACGACTAG	FM686	8	F	10	M-686
HI.4765.008.Oat-Plate8	1	AACCGAGA	FM687	8	F	11	M-687
HI.4765.008.Oat-Plate8	1	GTGAGGGT	FM688	8	F	12	M-688
HI.4765.008.Oat-Plate8	1	CGAT	FM689	8	G	1	M-689
HI.4765.008.Oat-Plate8	1	CTAGG	FM690	8	G	2	M-690
HI.4765.008.Oat-Plate8	1	GGTGT	FM691	8	G	3	M-691
HI.4765.008.Oat-Plate8	1	TAATA	FM692	8	G	4	M-692
HI.4765.008.Oat-Plate8	1	CCACAA	FM693	8	G	5	M-693
HI.4765.008.Oat-Plate8	1	CATCGT	FM694	8	G	6	M-694
HI.4765.008.Oat-Plate8	1	TGGCTA	FM695	8	G	7	M-695
HI.4765.008.Oat-Plate8	1	GTCGATT	FM696	8	G	8	M-696
HI.4765.008.Oat-Plate8	1	CGGTAGA	FM697	8	G	9	M-697
HI.4765.008.Oat-Plate8	1	TAGCATGG	FM698	8	G	10	M-698
HI.4765.008.Oat-Plate8	1	ACAGGGA	FM699	8	G	11	M-699
HI.4765.008.Oat-Plate8	1	TATCGGGA	FM700	8	G	12	M-700
HI.4765.008.Oat-Plate8	1	GTAA	FM702	8	H	1	M-702
HI.4765.008.Oat-Plate8	1	ACAAA	FM703	8	H	2	M-703
HI.4765.008.Oat-Plate8	1	AGGAT	FM704	8	H	3	M-704
HI.4765.008.Oat-Plate8	1	TACAT	FM705	8	H	4	M-705
HI.4765.008.Oat-Plate8	1	CTTCCA	FM706	8	H	5	M-706
HI.4765.008.Oat-Plate8	1	CGCGGT	FM707	8	H	6	M-707
HI.4765.008.Oat-Plate8	1	TATTTTT	FM708	8	H	7	M-708
HI.4765.008.Oat-Plate8	1	AACGCCT	FM717	8	H	8	Blank

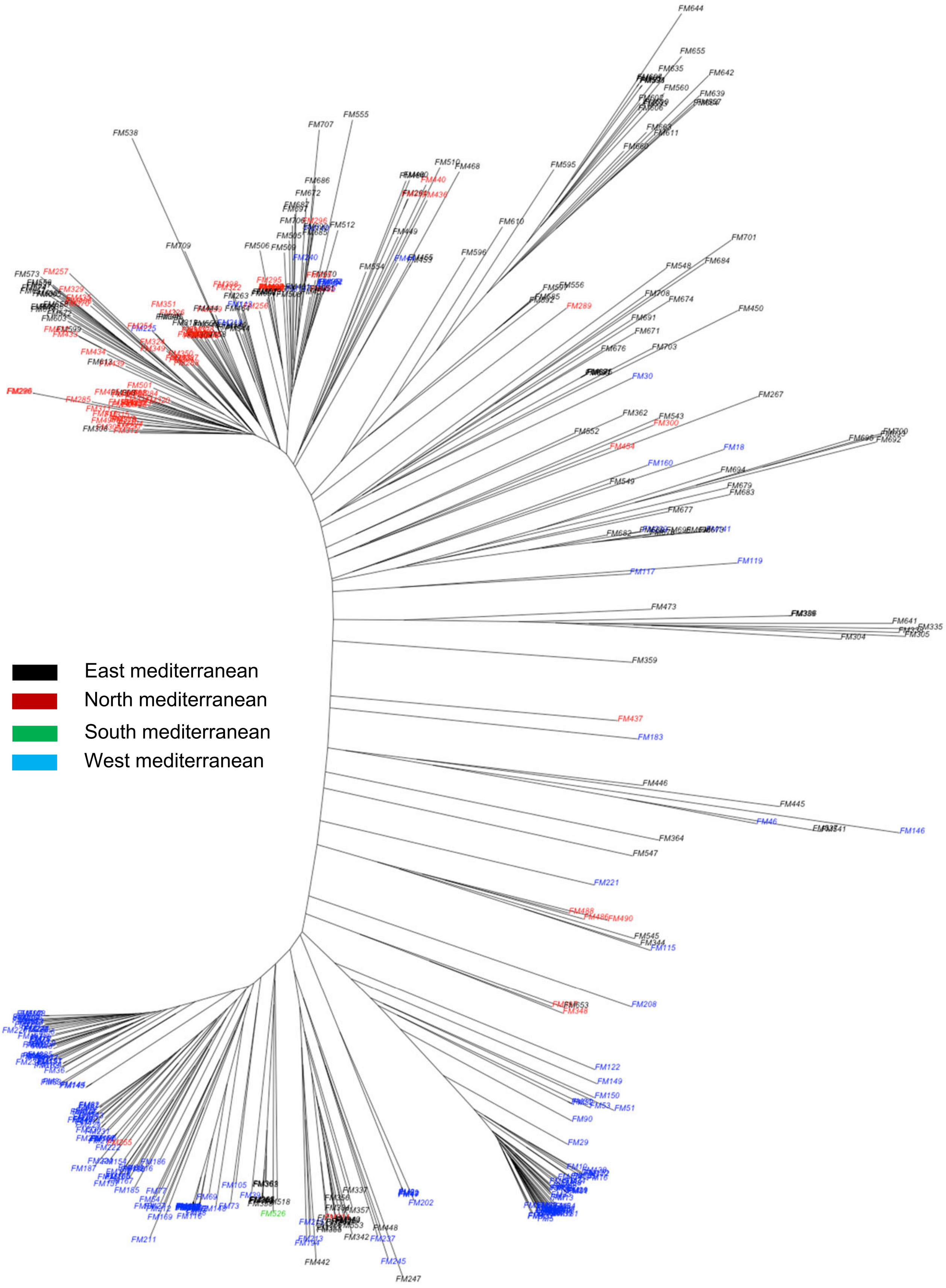

Online Resource 6 UPGMA dendogram of the white oat accessions. The phylogenetic map was based on DICE distance for the 17288 polymorphic SNP markers. Color of the accession numbers corresponded to the different regions of origin. Black = East Mediterranean; Red= North Mediterranean; Green = South Mediterranean; Blue= West mediterranean

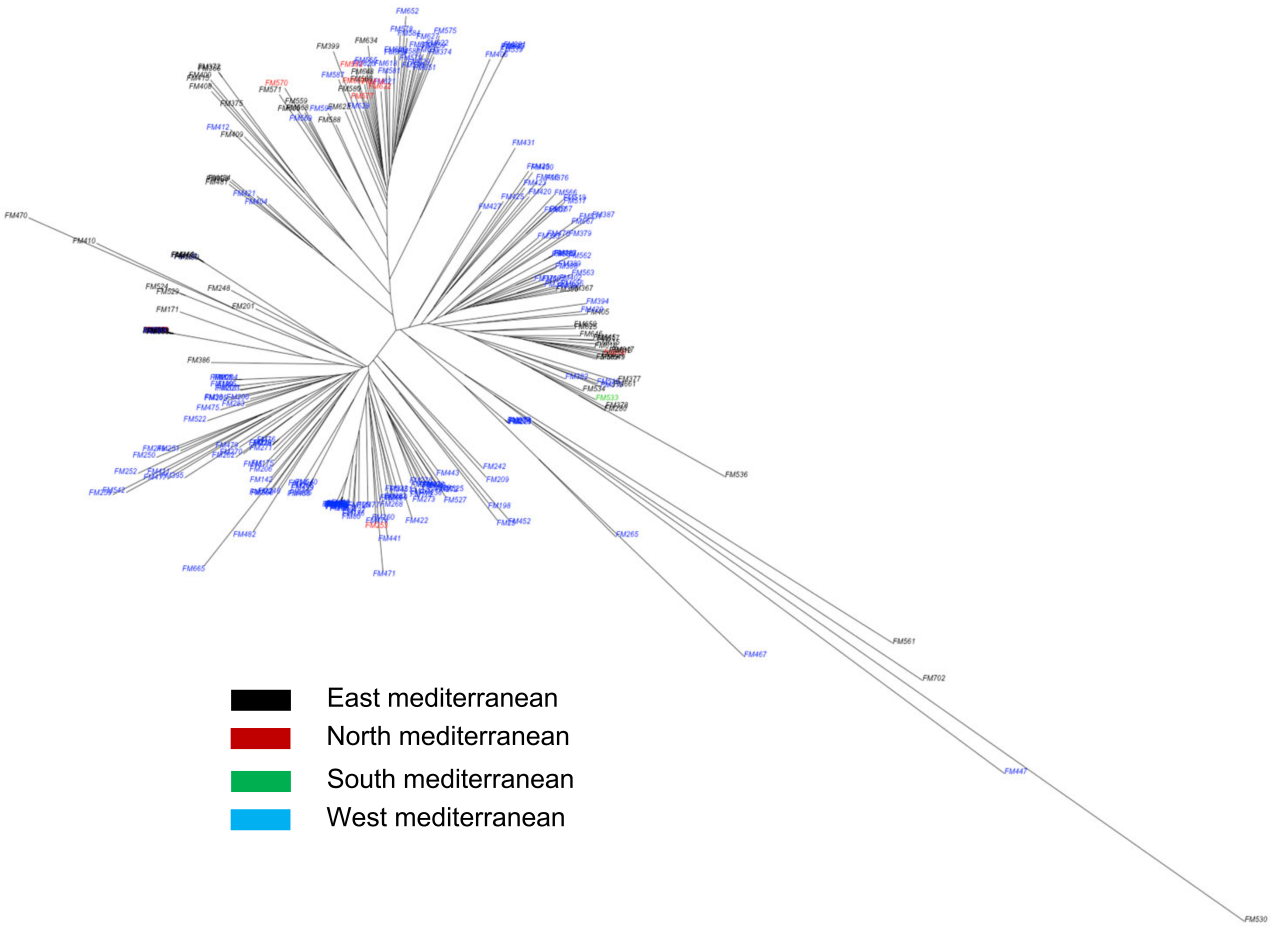

Online Resource 7 UPGMA dendogram of the red oat accessions. The phylogenetic map was based on DICE distance for the 17288 polymorphic SNP markers. Color of the accession numbers corresponded to the different regions of origin. Black = East Mediterranean; Red= North Mediterranean; Green = South Mediterranean; Blue= West mediterranean

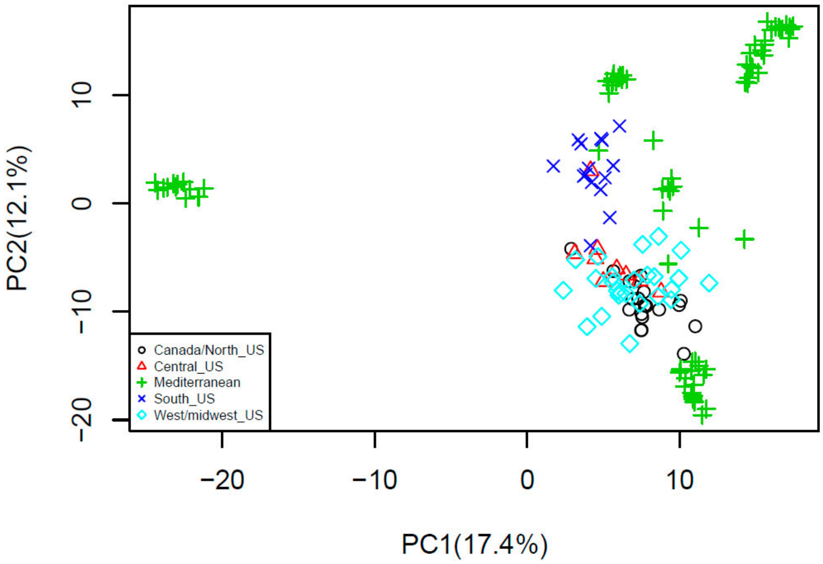

Online Resource 8. Scatterplot of Principal Component Analysis scores of components 1 and 2 of a random downsample of Mediterranean oats of similar size than North American oat accessions based on 20493 polymorphic SNP markers

Total accessions

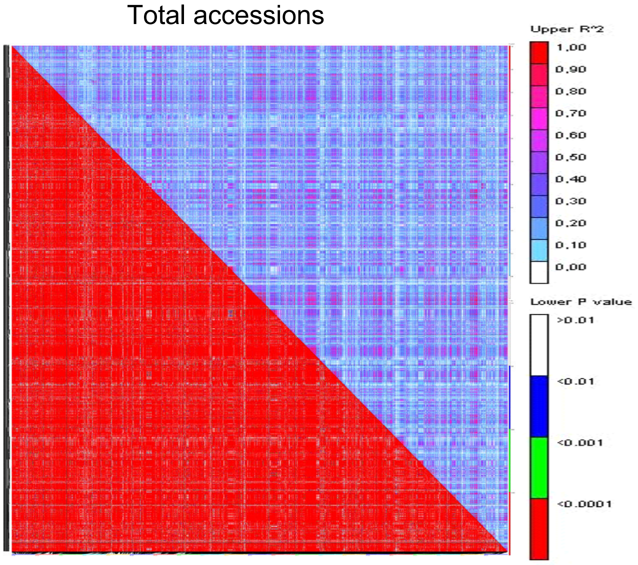

Markers pairs in significant LD = 9.298.006
Significant markers pairs $R^2 < 0.1$ = 5.773.494

subsp. *sativa*

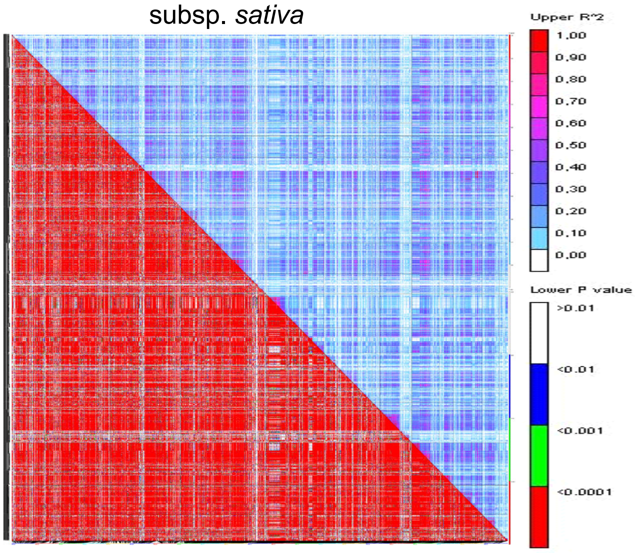

Markers pairs in significant LD = 5.286.730
Significant markers pairs $R^2 < 0.1$ = 2.869.144

subsp. *byzantina*

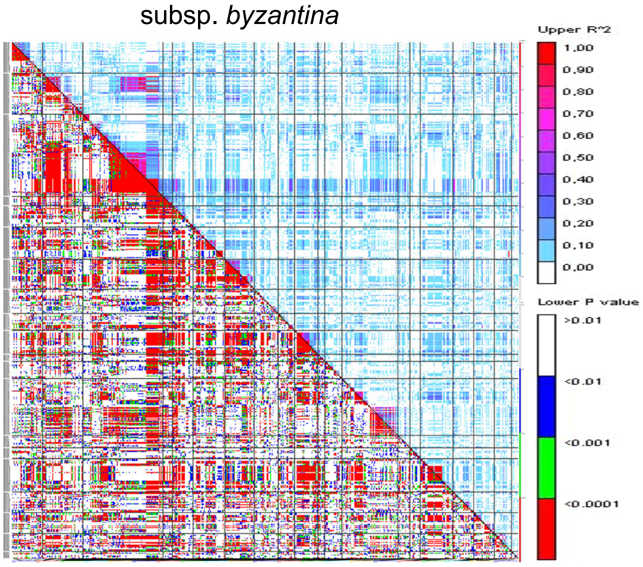

Markers pairs in significant LD = 174.220
Significant markers pairs $R^2 < 0.1$ = 99.327

Online Resource 9 Linkage disequilibrium matrix for A. *sativa* and A. *byzantina* subpopulations. The matrix display the r^2 values for each pair-wise polymorphic sites above the diagonal and the corresponding *p*-values from 1000 permutation test below the diagonal

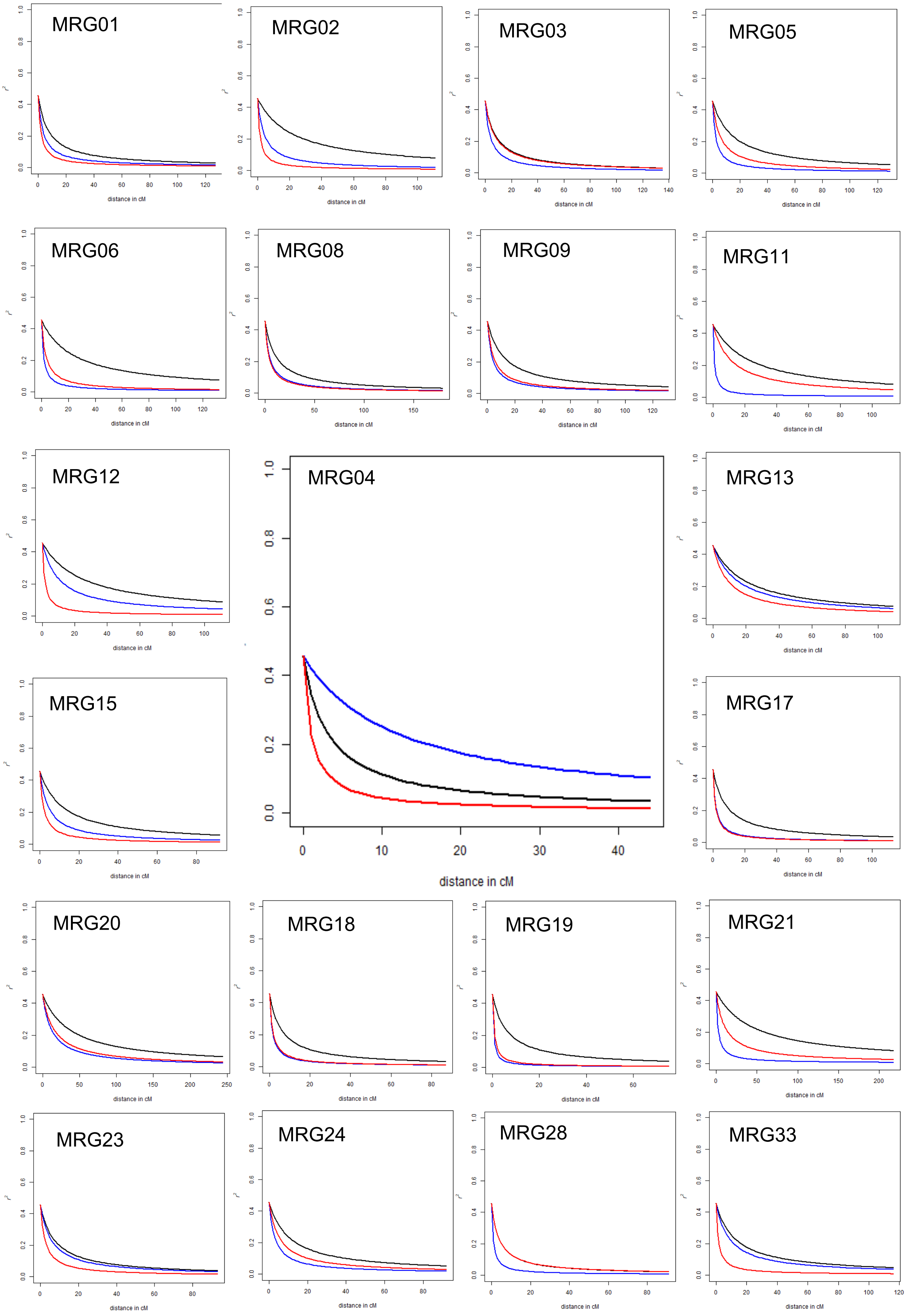

Online Resource 10 Linkage disequilibrium decay by distance across subspecies *sativa* (blue line), *byzantine* (red line) and both subspecies (black line) in the different oat linkage groups.

Online Resource 11 Potential candidate genes of significant markers in a recently released pseudomolecule assembly of hexaploid oat from the Canadian breeding line 'OT3098' (available July 2020 at https://wheat.pw.usda.gov/GG3/node/922) using the function BlastX of the BLAST algorithm (Altschul et al., 1990). Blastx 2.2.30.

Marker	Linckage group	Chr	Nearby gene	Gene/marker distance (kbp)	Position relative to marker	Results BLASTn NCBI					
						**Description**	**Species**	**E-value**	**Cov(%)**	**Ident(%)**	**Accession number**
avgbs_80864.1	Mrg13	2C	Pepsico2_Contig14999	61,52	Downstream	NAC transcription factor	*H. vulgare*	3E-115	59	78,09	FR821738.1
			Pepsico1_Contig141	143,64	Downstream	30S ribosomal protein 3, chloroplastic	*B. distachyon*	2E-142	20	87,53	XM_003579859.4
			Pepsico2_Contig14924	0,99	Upstream	Cyclic pyranopterin monophosphate synthase, mitochondrial (CNX3)	*B. distachyon*	0,0	13	89,33	XM_024456111.1
avgbs_cluster_4923.1	Mrg01	1D	Pepsico2_Contig18126	207,033	Downstream	Pectin acetylesterase 9	*B. distachyon*	2E-70	20	90,32	XM_024460397.1
			Pepsico2_Contig19827	305,37	Downstream	Proline dehydrogenase 2, mitochondrial-like	*A. tauschii*	0,0	47	94,16	XM_020328890.1
			TRINITY_DN23963_c0_g1_i2	554,62	Downstream	GDSL esterase/lipase At5g55050-like	*B. distachyon*	4E-36	9	85,03	XM_003559665.3
			Pepsico2_Contig19639	528,172	Upstream	Branched-chain-amino-acid aminotransferase 5, chloroplastic-like	*A.tauschii*	1E-63	12	92,31	XM_020325288.1
avgbs_cluster_1918.1	Mrg08	2D	TRINITY_DN43677_c0_g2_i2	39.850	Downstream	Clone B1 GASR7 gene	*T.aestivum*	2E-73	20	94,21	KJ000053.1
			TRINITY_DN23097_c0_g1_i1	194.168	Downstream	Transcription factor TGAL3-like	*A. tauschii*	2E-94	33	92,16	XM_020299922.1
			Pepsico1_Contig39146	5.946	Upstream	Autophagy-related 2	*A. tauschii*	0,0	50	88,43	XM_020305375.1

Note: GBS positions were declared when an exact match of a 64-base tag-level haplotype belonging to a given locus was found at one and only one genome position
